# Epidemiology of taeniosis/cysticercosis in Europe, a systematic review: Western Europe

**DOI:** 10.1186/s13071-017-2280-8

**Published:** 2017-07-21

**Authors:** Minerva Laranjo-González, Brecht Devleesschauwer, Chiara Trevisan, Alberto Allepuz, Smaragda Sotiraki, Annette Abraham, Mariana Boaventura Afonso, Joachim Blocher, Luís Cardoso, José Manuel Correia da Costa, Pierre Dorny, Sarah Gabriël, Jacinto Gomes, María Ángeles Gómez-Morales, Pikka Jokelainen, Miriam Kaminski, Brane Krt, Pascal Magnussen, Lucy J. Robertson, Veronika Schmidt, Erich Schmutzhard, G. Suzanne A. Smit, Barbara Šoba, Christen Rune Stensvold, Jože Starič, Karin Troell, Aleksandra Vergles Rataj, Madalena Vieira-Pinto, Manuela Vilhena, Nicola Ann Wardrop, Andrea S. Winkler, Veronique Dermauw

**Affiliations:** 1 IRTA, Centre de Recerca en Sanitat Animal (CReSA, IRTA-UAB), Campus de la Universitat Autònoma de Barcelona, Bellaterra, 08193 Barcelona, Spain; 2 Department of Public Health and Surveillance, Scientific Institute of Public Health (WIV-ISP), Brussels, Belgium; 3 Department of Veterinary and Animal Sciences, Faculty of Health and Medical Sciences, University of Copenhagen, Frederiksberg C, Denmark; 4 Departament de Sanitat i Anatomia Animals, Universitat Autònoma de Barcelona, Bellaterra, 08193 Barcelona, Spain; 5 Veterinary Research Institute, HAO-DEMETER, Campus Thermi, 57001 Thessaloniki, Greece; 6 Centre for Global Health, Department of Neurology, Technical University Munich, Ismaninger Strasse 22, 81675 Munich, Germany; 7 Centre for Global Health, Department of Community Medicine and Global Health, Institute of Health and Society, University of Oslo, Kirkeveien 166, 0450 Oslo, Norway; 8 Divisão de Proteção Veterinária e Pecuária, Direção de Serviços de Alimentação e Veterinária, Direção Regional de Agricultura, Secretaria Regional de Agricultura e Pescas, Av. Arriaga, 21 Edifício Golden, 3° Andar, 9000-690 Funchal, Portugal; 9 Institute for Acute Neurology and Stroke, Academic Teaching Hospital Feldkirch, Feldkirch, Austria; 10 Department of Veterinary Sciences, School of Agrarian and Veterinary Sciences, University of Trás-os-Montes e Alto Douro, 5000-801 Vila Real, Portugal; 11 Center for Parasite Biology and Immunology, National Institute of Health Dr. Ricardo Jorge, Rua Alexandre Herculano 321, 4000-055 Porto, Portugal; 12 Center for the Study of Animal Science (CECA), Institute for Agricultural and Agro-Alimentary Science and Technology (ICETA), University of Porto, Porto, Portugal; 13 Department of Virology, Parasitology and Immunology, Faculty of Veterinary Medicine, Ghent University, Merelbeke, Belgium; 14 Department of Biomedical Sciences, Institute of Tropical Medicine, Antwerp, Belgium; 15 Department of Veterinary Public Health and Food Safety, Faculty of Veterinary Medicine, Ghent University, Ghent, Belgium; 16 National Institute for Agrarian and Veterinary Research, Oeiras, Portugal; 17 European Union Reference Laboratory for Parasites, Istituto Superiore di Sanità, 00161 Rome, Italy; 18 Faculty of Veterinary Medicine, University of Helsinki, P.O. Box 66, 00014 Helsinki, Finland; 19 Laboratory of Parasitology, Department of Bacteria, Fungi & Parasites, Infectious Disease Preparedness, Statens Serum Institut, Artillerivej 5, DK-2300 Copenhagen S, Denmark; 20 Department of Basic Veterinary Sciences and Population Medicine, Institute of Veterinary Medicine and Animal Science, Estonian University of Life Sciences, Kreutzwaldi 62, 51014 Tartu, Estonia; 21 Department of Neurology, Klinikum rechts der Isar, Technical University Munich, Ismaninger Straße 22, 81675 Munich, Germany; 22 Institute for Microbiology and Parasitology, Veterinary Faculty, University of Ljubljana, Gerbičeva 60, 1000 Ljubljana, Slovenia; 23 Department of Immunology and Microbiology, Centre for Medical Parasitology, University of Copenhagen, Copenhagen, Denmark; 24 Department of Veterinary and Animal Sciences, section for Parasitology and Aquatic Diseases, Faculty of Health and Medical Sciences, University of Copenhagen, Copenhagen, Denmark; 25 Department of Food Safety and Infection Biology, Faculty of Veterinary Medicine, Norwegian University of Life Sciences, Adamstuen Campus, 0033 Oslo, Norway; 26 Department of Neurology, NICU Medical University Innsbruck, Anichstrasse 35, A-6020 Innsbruck, Austria; 27 Institute of Health and Society (IRSS), Université catholique de Louvain, Brussels, Belgium; 28 Institute of Microbiology and Immunology, Faculty of Medicine, University of Ljubljana, Ljubljana, Slovenia; 29 Clinic for reproduction and large animals - section for ruminants, Veterinary faculty, University of Ljubljana, Ljubljana, Slovenia; 30 National Veterinary Institute, SE-751 89 Uppsala, Sweden; 31 CECAV - Animal and Veterinary Research Centre, UTAD, Quinta de Prados, 5000-801 Vila Real, Portugal; 32 Instituto de Ciências Agrárias e Ambientais Mediterrânicas (ICAAM), Universidade de Évora, Évora, Portugal; 33 Geography and Environment, University of Southampton, Highfield Campus, Southampton, England SO17 1BJ UK

**Keywords:** *Taenia solium*, *Taenia saginata*, Taeniasis, Neurocysticercosis, Porcine cysticercosis, Bovine cysticercosis

## Abstract

**Background:**

*Taenia solium* and *Taenia saginata* are zoonotic parasites of public health importance. Data on their occurrence in humans and animals in western Europe are incomplete and fragmented. In this study, we aimed to update the current knowledge on the epidemiology of these parasites in this region.

**Methods:**

We conducted a systematic review of scientific and grey literature published from 1990 to 2015 on the epidemiology of *T. saginata* and *T. solium* in humans and animals. Additionally, data about disease occurrence were actively sought by contacting local experts in the different countries.

**Results:**

Taeniosis cases were found in twelve out of eighteen countries in western Europe. No cases were identified in Iceland, Ireland, Luxembourg, Norway, Sweden and Switzerland. For Denmark, Netherlands, Portugal, Slovenia, Spain and the UK, annual taeniosis cases were reported and the number of detected cases per year ranged between 1 and 114. Detected prevalences ranged from 0.05 to 0.27%, whereas estimated prevalences ranged from 0.02 to 0.67%. Most taeniosis cases were reported as *Taenia* spp. or *T. saginata*, although *T. solium* was reported in Denmark, France, Italy, Spain, Slovenia, Portugal and the UK. Human cysticercosis cases were reported in all western European countries except for Iceland, with the highest number originating from Portugal and Spain. Most human cysticercosis cases were suspected to have acquired the infection outside western Europe. Cases of *T. solium* in pigs were found in Austria and Portugal, but only the two cases from Portugal were confirmed with molecular methods. Germany, Spain and Slovenia reported porcine cysticercosis, but made no *Taenia* species distinction. Bovine cysticercosis was detected in all countries except for Iceland, with a prevalence based on meat inspection of 0.0002–7.82%.

**Conclusions:**

Detection and reporting of taeniosis in western Europe should be improved. The existence of *T. solium* tapeworm carriers, of suspected autochthonous cases of human cysticercosis and the lack of confirmation of porcine cysticercosis cases deserve further attention. Suspected cases of *T. solium* in pigs should be confirmed by molecular methods. Both taeniosis and human cysticercosis should be notifiable and surveillance in animals should be improved.

**Electronic supplementary material:**

The online version of this article (doi:10.1186/s13071-017-2280-8) contains supplementary material, which is available to authorized users.

## Background

*Taenia solium* and *Taenia saginata* are zoonotic tapeworm species that cause taeniosis in humans (definitive host) and cysticercosis in pigs and cattle (intermediate hosts), respectively. Humans can also acquire cysticercosis after accidentally ingesting *T. solium* eggs. Cysticerci in humans often establish in the central nervous system causing neurocysticercosis (NCC) [1].

Human taeniosis causes few or no symptoms [2] although it can cause psychological stress [3]. Animal cysticercosis is normally asymptomatic, particularly if infections are light. However, cases are responsible for substantial economic losses to the meat sector [4]. NCC may be asymptomatic, but it can cause neurological manifestations such as seizures, headaches, focal neurological deficits, signs of increased intracranial pressure and deaths [5, 6] and is a leading cause of acquired epilepsy in endemic areas [7].

*Taenia solium* is considered to be endemic in parts of Asia, sub-Saharan Africa and South and Central America [8]. In Europe, industrialisation of pig rearing systems and improved sanitation are believed to have eliminated the parasite [9, 10]. However, gaps regarding the true endemicity status of *T. solium* in Europe still remain [10]*.* According to a map on *T. solium* endemicity, recently updated by the World Health Organization [8], some countries in western Europe still have some pig herds at risk of *T. solium* transmission. Furthermore, the epidemiological situation in eastern Europe is unclear since there are countries classified as endemic, with some pig herds at risk, and countries from which data are lacking [8]. In addition, *T. solium* in humans has been emerging as a public health concern in Europe due to the increased number of diagnosed NCC cases in recent decades. These have been linked to increased travels and migratory movements towards and from endemic countries [11–14].

*Taenia saginata* is distributed worldwide [15], and has been found in cattle in countries of western and eastern Europe. However, the available data are limited and often of low quality [16]. Data on taeniosis due to *T. saginata* are scarce, and among the data that do exist, its prevalence is sometimes estimated from the sales of anthelmintic drugs [17].

Taeniosis and human cysticercosis are not notifiable in Europe as stated by Gäbriel et al. [9] and therefore it is difficult to assess the epidemiology of these zoonoses in the region. Detection and reporting of animal cysticercosis cases is mainly based on official meat inspection. Porcine cysticercosis has to be notified to the World Organisation for Animal Health (OIE), but there is no mandatory reporting for bovine cysticercosis. Despite the European Directive 2003/99/EC [18] that recommends monitoring animal cysticercosis according to the epidemiological situation, few countries report these cases [16, 19].

Based on the need for useful estimates for taeniosis/cysticercosis surveillance and control activities, as well as to advance the knowledge and awareness of these zoonotic disease complexes, the aim of this review was to update and compile the current knowledge on the epidemiology of *T. solium* and *T. saginata* in western Europe (both in humans and animals). This review is one of two systematic reviews: the present review covers western Europe and a second review will cover eastern Europe.

## Methods

### Study design

We conducted a systematic review supplemented by a search of local and unpublished sources for information on the occurrence, prevalence, incidence and the geographical distribution of human and animal *T. saginata* and *T. solium* infections in western Europe published from 1990 to 2015. This area was defined, based on gross domestic product/gross national income (GDP/GNI) and regional proximity, as including the following countries: Austria, Belgium, Denmark, Finland, France, Germany, Iceland, Ireland, Italy, Luxembourg, Norway, the Netherlands, Portugal, Slovenia, Spain, Sweden, Switzerland and the United Kingdom; and excluding overseas territories and mini-states (e.g. Liechtenstein).

### International databases

We searched the following online international databases: PubMed, ISI Web of Knowledge, CABDirect, OAIster and OpenGrey for all published data on the topic and followed the PRISMA (Preferred Reporting Items for Systematic Reviews and Meta-Analyses) guidelines for reporting systematic reviews (Additional file 1: Table S1). The following search phrase was used: (cysticerc* OR cisticerc* OR neurocysticerc* OR neurocisticerc* OR “C. bovis” OR “C. cellulosae” OR taenia* OR tenia* OR saginata OR solium OR taeniosis OR teniosis OR ténia OR taeniid OR cysticerque) AND (Austria OR België OR Belgiën OR Belgique OR Belgium OR Denmark OR Deutschland OR Éire OR England OR España OR Finland OR France OR Germany OR Iceland OR Ireland OR Ísland OR Italia OR Italy OR Luxembourg OR Nederland OR Netherlands OR Norway OR Österreich OR Portugal OR Schweiz OR Scotland OR Slovenia OR Slovenija OR Spain OR Suisse OR Svizzera OR Sweden OR Switzerland OR United Kingdom OR Wales). The databases were searched for papers published from 1st January 1990 up to 1st December 2015 (even if containing data older than 1990). Papers were excluded if at least one of the following criteria were met: (i) studies did not concern *T. saginata* and/or *T. solium*; (ii) studies did not report data from within the specified area; (iii) studies published before 1990 or after 1st December 2015; (iv) studies reported results outside the scope of the study questions (including general reviews on the topic). Papers were initially screened for eligibility primarily based on title and abstract, and, if necessary, the full paper was assessed. If the full text was not available, relevant data provided in the abstract were included. From each eligible document, data were collected in predefined tables.

### Local sources

We distributed country sheets (Additional file 2) to members of the European Network on Taeniosis/Cysticercosis (CYSTINET, COST Action TD1302) and other experts, requesting them to list relevant national journals, epidemiological bulletins, MSc/PhD dissertation databases, national institutes, and registries, and to translate relevant search terms. Due to ethical constraints, unpublished hospital or laboratory data were requested at an aggregated level. In addition, we searched for relevant records in meeting proceedings of CYSTINET and the European Network for Foodborne Parasites (Euro-FBP, COST Action FA1408). Finally, we explored the references listed in recent topic-specific reviews [12–14, 16] to identify any additional eligible documents. We applied the same inclusion and exclusion criteria and followed the same data collection approach for all eligible sources. Personal communications received after 1st of December 2015 were allowed to be included when describing data from within the study period.

### Data collection and analyses

Three independent reviewers (VD, MLG, CT) performed the data collection. For data analysis, cases reported as case reports providing information on individual characteristics of the patient were defined as individual cases. Cases provided at aggregated level with no individual information were defined as aggregated cases. Predefined tables summarising individual cases included year of diagnosis, age, gender, country of origin or nationality, and reported risk factors, and reference (i.e. author and publication year). Tables summarising aggregated cases or prevalence included country, level of data collection (e.g. national/regional), timeframe, number of cases (or prevalence), *Taenia* species, risk factors (e.g. immigration/travel history) if available, and reference.

For description of risk factors, we applied the following definitions: (i) Endemic region: Asia, Africa, South and Central America (including Caribbean islands), and eastern European countries; (ii) Immigrant: any person born in or native from an endemic region, or reported to have moved from an endemic region; (iii) Travelled/stayed in endemic region: having travelled, stayed, or resided in an endemic region reported in their epidemiological history; (iv) No history of travels to endemic areas or immigration (autochthonous): information on risk factors provided, but no history of travel/immigration (outside western Europe) is reported.

In those cases where the existence of duplicates was probable (e.g. cases included in two retrospective studies on the same area/hospital, covering overlapping time periods, cases diagnosed in the same hospital in the same timeframe but reported in different sources, etc.) cases were only presented once. Descriptive analyses and graphics were performed in Excel and the R software environment for statistical computing (R Core Team, 2016).

## Results

### Search results

**Fig. 1:**
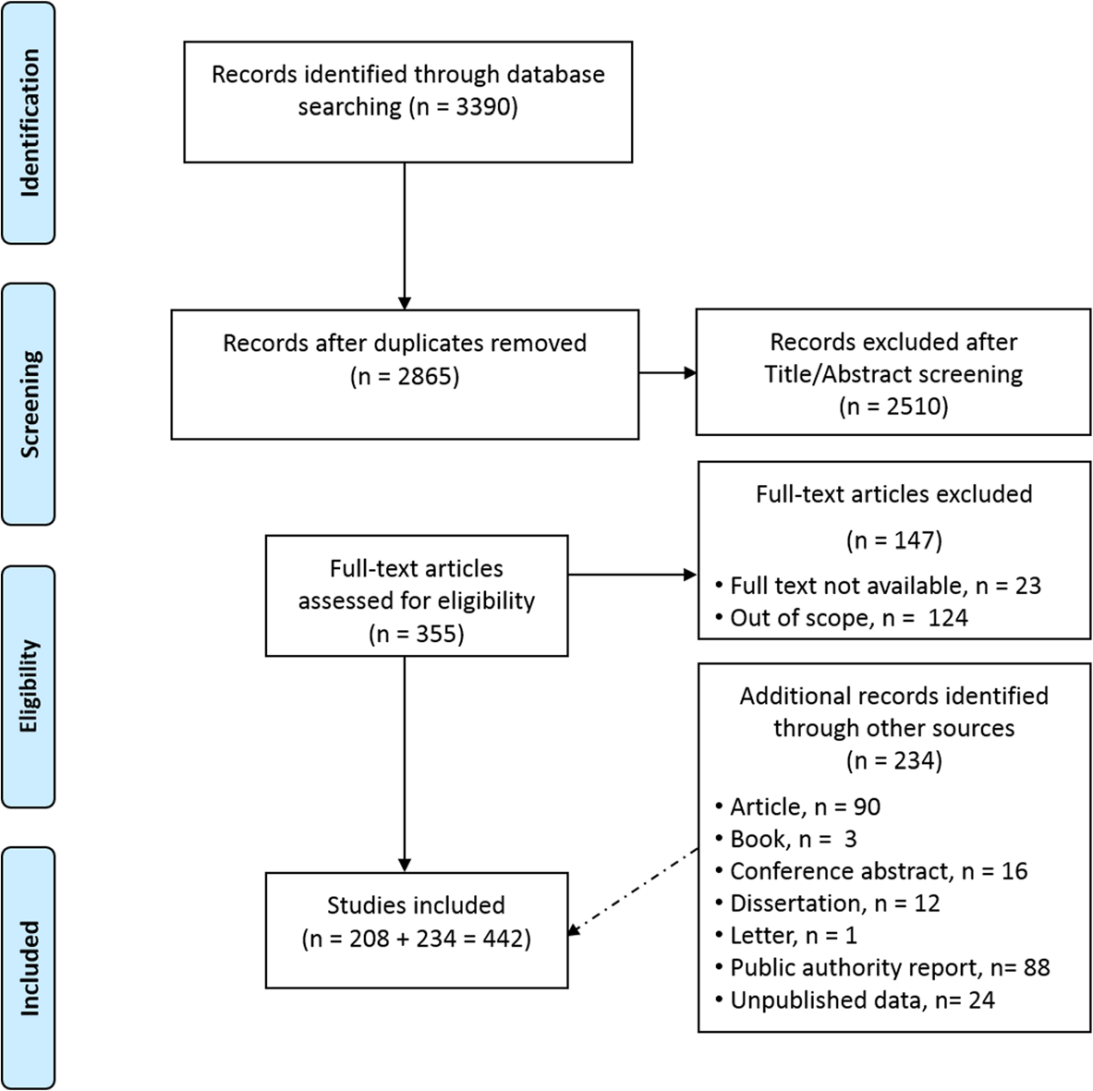
Flow diagram of the search strategy steps

The steps followed in the search strategy are presented in Fig. 1. A total of 442 relevant references were identified and included in the review: 208 were retrieved through online international databases (Additional file 3: Table S2) and 234 were made available through local sources (Additional file 4: Table S3).

**Fig. 2:**
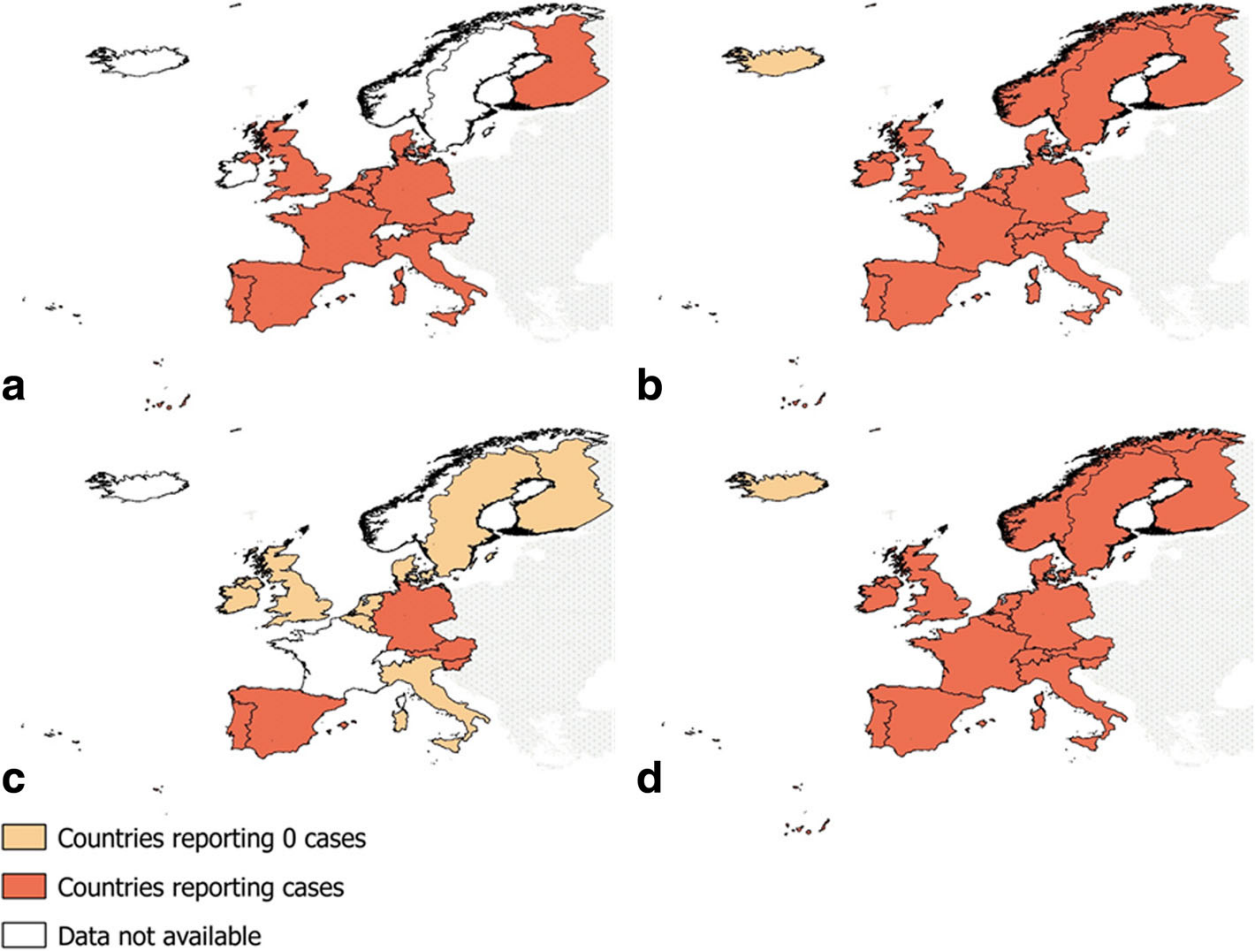
Summary of identified data on human taeniosis and cysticercosis in western Europe (1990–2015). **a** Taeniosis. **b** Human cysticercosis. **c** Porcine cysticercosis. **d** Bovine cysticercosis

The countries for which we identified relevant data or cases of *T. saginata* or *T. solium* in humans or animals are shown in Fig. 2. Data were retrieved from peer-reviewed papers, governmental and scientific reports (e.g. EFSA reports), epidemiological bulletins, dissertations, conference abstracts, and from sources providing unpublished data (e.g. registries and personal communications).

#### Taeniosis

We identified 86 sources providing unique information for twelve countries: 21 records reporting 22 individual cases and 65 providing information on aggregated cases or prevalence. For Finland, the only information found indicated that a handful of taeniosis cases are diagnosed in HUSLAB yearly (*T. solium* being less common than *T. saginata*) [20, 21]. No reports of taeniosis could be found for Iceland, Ireland, Luxembourg, Norway, Switzerland and Sweden.

#### Taeniosis case reports

**Fig. 3:**
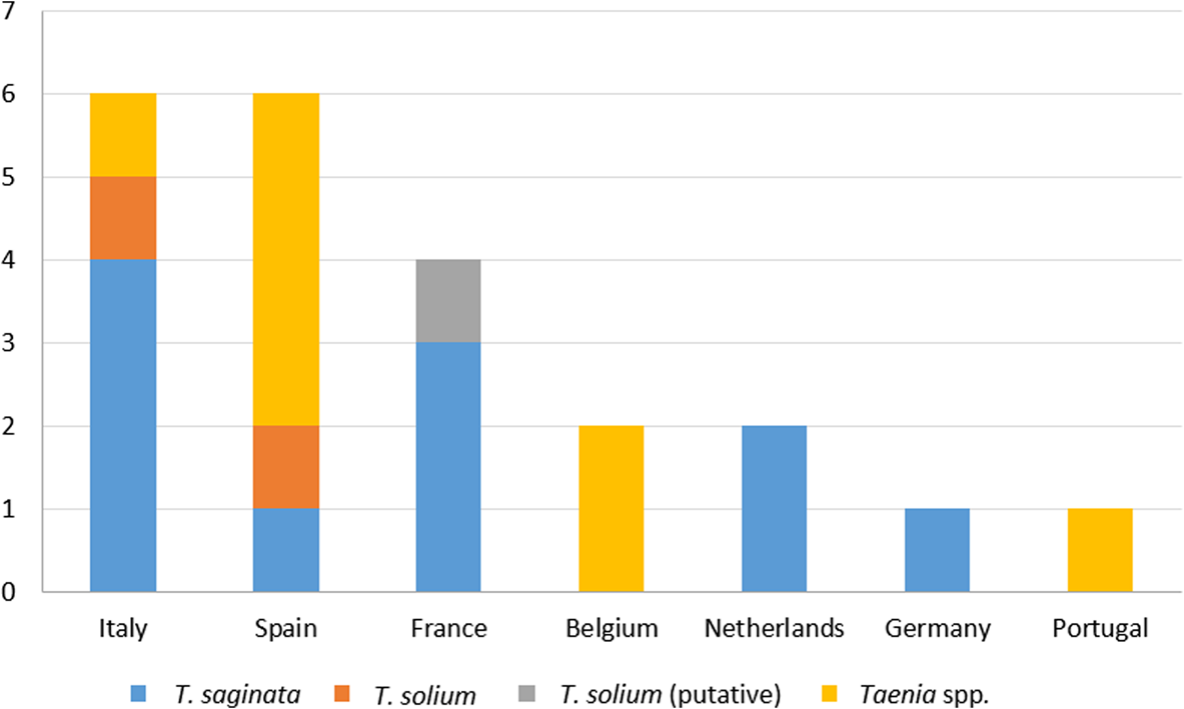
Number of identified taeniosis cases in case reports in western Europe (1990–2015)

In total, 22 individual cases were reported in seven countries (Additional file 5: Table S4). Almost all were reported as *T. saginata* (11 cases) or *Taenia* spp. (8 cases, one of them suspected to be *T. saginata*) (Fig. 3). Two case reports of *T. solium* were found, one in Spain (in a 19 year-old Spanish woman who had consumed raw pork) and one in Italy (a post-mortem diagnosis in a 26-year old farmer in 1985). A *T. solium* case was suspected in Corsica (France) in a 55 year-old woman who had consumed a Corsican traditional dish made with uncooked pig intestine [22] although Galán-Puchades & Fuentes [23] later suggested that *Taenia asiatica* could have been the causative agent. None of the *T. solium* case reports provided details on how the species identification was achieved.

In half of the cases, consumption of raw meat was mentioned as a risk factor. It was not mentioned whether the taeniosis cases could be autochthonous or imported except for one patient who had recently returned from a prolonged stay in Ivory Coast.

#### Aggregated taeniosis cases

Aggregated taeniosis cases were obtained from authorities’ reports, epidemiological bulletins, or national registries (Additional file 5: Table S5) and from hospitals/laboratories and epidemiological studies (Additional file 5: Table S6).

**Fig. 4:**
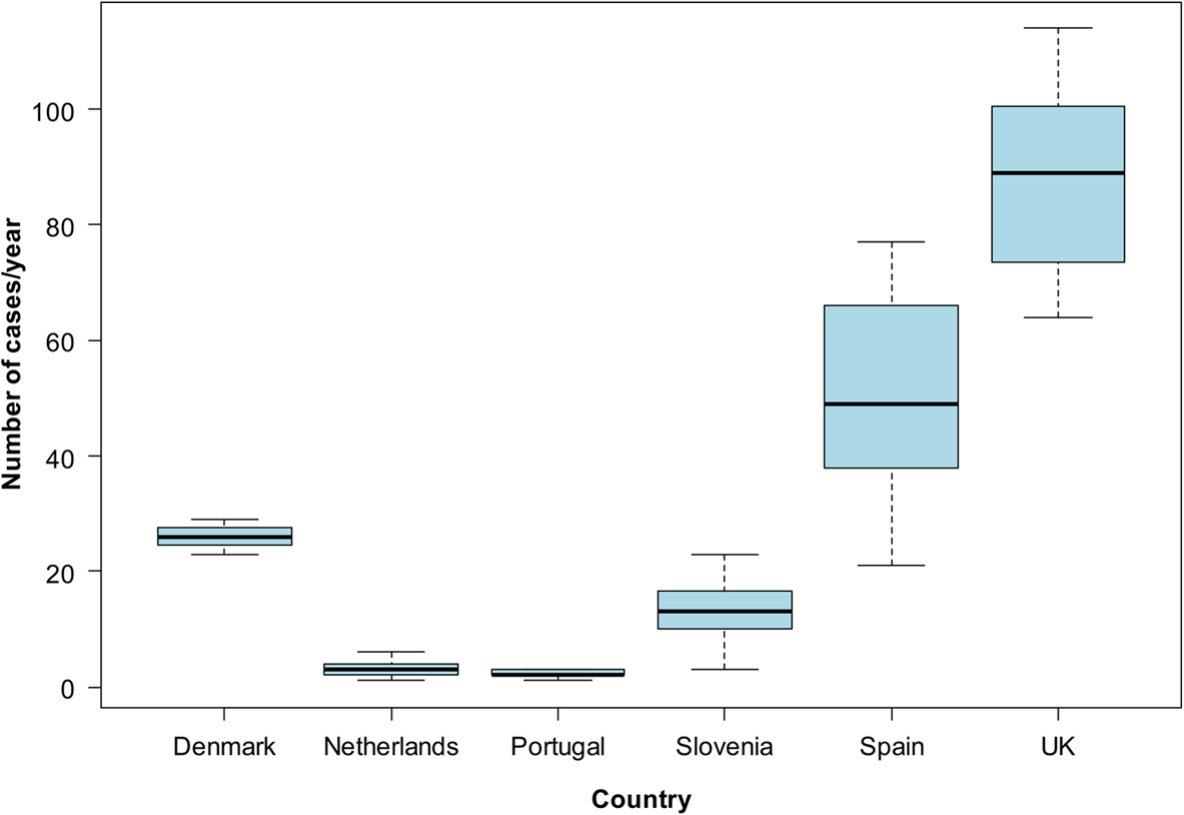
Number of aggregated taeniosis cases/year reported in authorities’ reports, epidemiological bulletins and national registries in western Europe (1990–2015). Data from Portugal do not include the autonomous regions of Madeira and Azores

Data from authorities’ reports, epidemiological bulletins or national registries were available for six countries covering different years. The number of cases reported per year by each country was variable, with the UK and Spain reporting the highest number of annual cases (Fig. 4). Most cases were reported as *Taenia* spp. or *T. saginata*; however, eight *T. solium* cases in Spain (reported in different years between 2001 and 2008), eight in Portugal (reported in different years between 2000 and 2011), five in Slovenia (detected in different years between 1997 and 2011), and two in the UK (one case reported in 2002 and another in 2003) were identified. According to Hill et al. [24] around 98% of the cases recorded by the Health Protection Agency in the UK in the last years were *T. saginata*. For most cases, no information was available in relation to nationality, risk factors, or sources of infection. Of all aggregated taeniosis cases reported in the UK, one case reported having eaten raw beef and 46 cases were connected with overseas travels. The total number of cases per *Taenia* species and country is shown in the Additional file 5: Table S5.

**Fig. 5:**
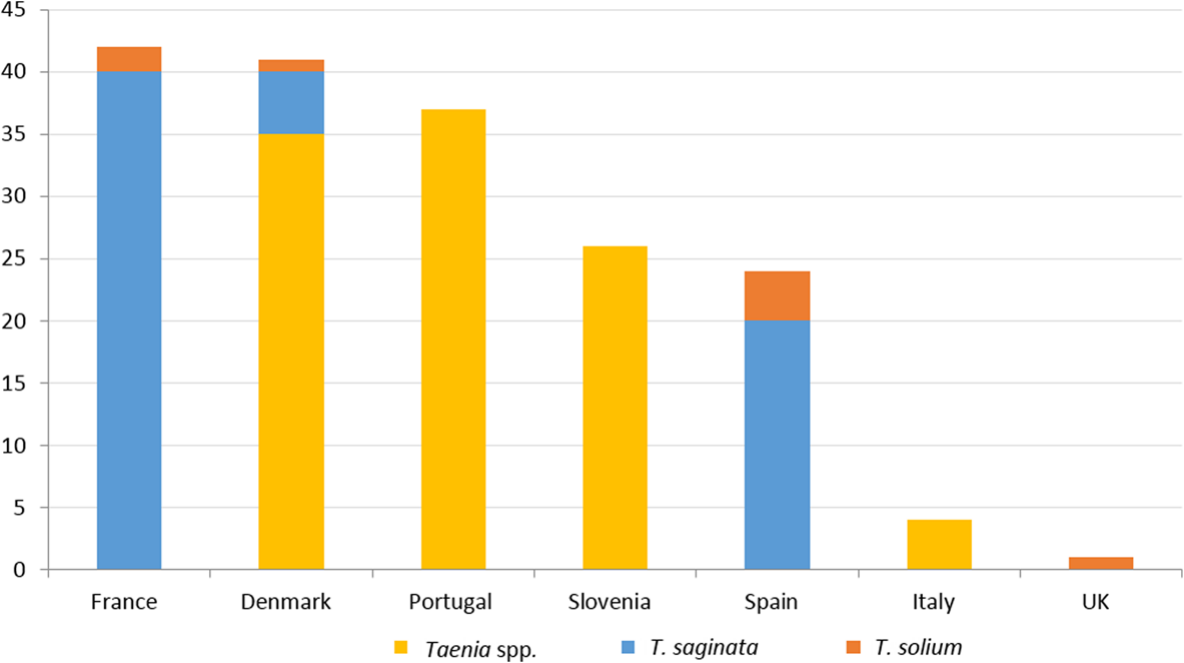
Number of aggregated taeniosis cases reported at hospital/laboratory level in western Europe (1990–2015). Data for Portugal correspond to the Autonomous Region of Madeira

Aggregated taeniosis cases identified from laboratory/hospital data and epidemiological studies (e.g. retrospective studies in hospitals) were identified for seven countries (Fig. 5). Further details are presented in the Additional file 5: Table S6.

#### Taeniosis prevalence data

Prevalence data were reported in regional epidemiological studies conducted at hospital or laboratory level. These studies were conducted in five countries at different time periods and reported *T. saginata* or *Taenia* spp*.* prevalences ranging between 0.05 and 0.27% (Additional file 5: Table S7).

Based on anthelmintic drugs sales, several authors have estimated the number of taeniosis cases or prevalence in a given region or country (Additional file 5: Table S8). The estimated number of *Taenia* cases occurring annually in Belgium and France was 11,350 and 64,495, respectively [25, 26]. Estimated prevalences range from 0.02 to 0.67%, with the highest being reported in Germany (0.33–0.67%) and Belgium (0.35–0.46%) and the lowest in Denmark (0.02%) and Italy (0.02–0.04%). In France, based on the quantification of taeniid egg contents in sludge, Barbier et al. [27] deducted that *T. saginata* taeniosis prevalence in the Caen urban area ranged from 1.5 to 2.7% (1987–1989).

#### Human cysticercosis

We identified 243 relevant sources providing unique information on human cysticercosis in all 18 countries.

#### Human cysticercosis case reports

**Fig. 6:**
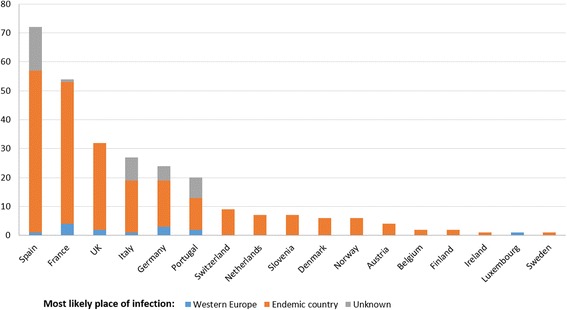
Number of identified human cysticercosis cases in case reports in western Europe (1990–2015)

A total of 275 individual cysticercosis cases were reported in 17 countries (Fig. 6). No case reports were identified for Iceland. Spain (72 cases) and France (54 cases) recorded the highest number of cases. The average number of cases published per year was 10.6 with 2014 being the year with the highest number (25) and 1997 the year with the lowest number (1) reported, respectively, among all 17 countries. The age of patients ranged from 2 to 94 years; 129 were female and 127 male (gender unknown in 19 cases).

Information on risk factors was reported in most cases (Additional file 5: Table S9). In 82% of cases the infection was probably acquired outside western Europe (61% due to immigration and 21% due to travels or stays in endemic regions). Among infected immigrants, the highest number of cases had emigrated from Latin America (77), followed by Asia (39) and Africa (35), whereas 15 cases originated from eastern Europe (e.g. Albania, Bosnia and Herzegovina, former Yugoslavia). For 5% of cases, the infection appeared to be acquired autochthonously (no travel/immigration history reported) (Table 1, Fig. 6). For the remaining cases (13%), there was no information on nationality or risk factors that could be linked with the infection.

**Table 1:** Suspected autochthonous human cysticercosis cases from case reports

Country	No. of cases	Background	Age (yrs)
France	4	1 case: had never left Europe 1 case: had never left metropolitan France 1 case: no history of travel to endemic areas 1 case: had never left France	44–69
Germany	3	3 cases: no history of travel to foreign countries	6–69
UK	2	1 case: no history of travelling outside Europe 1 case: lack of travel to endemic areas	3–21
Portugal	2	1 case: without relevant personal background 1 case: no history of travelling abroad	57–71
Italy	1	1 case: had never visited endemic areas for cysticercosis	61
Luxembourg (infection could have been acquired in Spain)	1	1 case: born in Spain, moved to Luxembourg 8 years prior diagnosis (annual visits to Spain)	20
Spain	1	1 case: without background of interest except for that he was a pig breeder	70

#### Aggregated human cysticercosis cases

Aggregated human cysticercosis cases were obtained from authorities’ reports or registries (Additional file 5: Table S10) and from hospital/laboratories or epidemiological studies (Additional file 5: Table S11).

Data from authorities’ reports and registries were available for six countries over different periods. The highest number of cases was reported in Spain, with 1702 hospitalised cases with diagnosis of cysticercosis at hospital discharge between 1997 and 2014 (range of 45–169 hospitalisations per year), following ICD-coding systems [28]; Portugal with 1120 hospitalised cysticercosis cases between 1993 and 2004 and 357 NCC hospitalised cases between 2006 and 2013 (mean of 45 cases per year) following ICD-coding systems [29, 30]; and Italy with 540 hospitalisations for cysticercosis between 2001 and 2010 (range of 40–53 per year) based on ICD-coding systems [31]. In Denmark, the national inpatient diagnosis register recorded 32 cases during 2012–2014 and in the Netherlands there were 24 hospitalisations with cysticercosis as primary diagnosis (following ICD codes) during 1986–1990. In Iceland, based on governmental reports there were no cases notified in 2013–2014 [32].

**Fig. 7:**
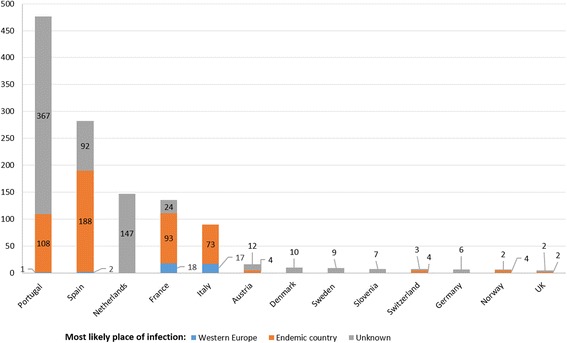
Number of aggregated human cysticercosis cases reported at hospital/laboratory level in western Europe (1990–2015)

Cases based on laboratory/hospital data or epidemiological studies were retrieved for 13 countries. The highest numbers of cysticercosis cases were diagnosed in Portugal (476) and Spain (282), followed by a lower number in the Netherlands (147), France (135) and Italy (90) (Fig. 7). Of these cases, 38 [diagnosed in France (18), Italy (17), Spain (2) and Portugal (1)] had most likely acquired the infection in western Europe based on the travel/immigration history reported. The 18 cases diagnosed in France were reported to have acquired the infection mainly in the Iberian Peninsula in 1978–1988. Further details are shown in Additional file 5: Table S11.

#### Porcine cysticercosis

We identified 39 relevant references providing unique information on 14 countries: 25 provided cases and 14 provided prevalence data. No information could be obtained for France, Iceland, Norway and Switzerland (Additional file 5: Table S12).

Based on the available information, no cases of porcine cysticercosis were identified during meat inspection at slaughter in Belgium, Denmark, Finland, Ireland, Italy, Luxembourg, the Netherlands, Sweden and the UK. According to public authorities, *T. solium* in pigs has not been reported for many years in the UK [33]. In Denmark, the last report of cysticerci in pork dated back to 1894 [34] and in Italy, according to Tamburrini et al. [35], porcine cysticercosis cases were only occasionally observed (e.g. in Basilicata) in the past.

Porcine cysticercosis was reported during meat inspection at slaughter in Austria, Germany, Portugal, Slovenia and Spain. Important to note, reports from Germany did not differentiate between cases of *Taenia hydatigena* and *T. solium* cysticercosis and Spain and Slovenia reported porcine cysticercosis with no further information on the causative species. Therefore, it is not possible to assess whether these cases were of public health importance. Slovenia notified only one case of porcine cysticercosis in 2007 (2007–2014), but it was not confirmed by any laboratory diagnostic method [36]. The reported prevalence in Germany ranged from 0 to 0.0023% (2009–2012). In Spain, the prevalence ranged from 0 to 0.20% in domestic pigs (1999–2014); 0.16 to 0.43% in home-slaughtered pigs (2011–2013), and 0 to 0.19% in wild boar (2009–2013).

In Extremadura (Spain), García Vallejo [37] analysed samples of 689 Iberian pigs raised on extensive breeding farms and could not identify any infected with *T. solium* cysticerci.

Austria was the only country where the veterinary authority had been annually (between 1998 and 2002) reporting cases of *T. solium* [reported as “*Cysticercus cellulosae*”: 10–40 cases/year (1999–2002); 0 cases in 1998] [38–42]. Most of these cases were described as light infections (65 light and 23 heavy infections during 1999–2002).

In Portugal, two confirmed cases of generalised cysticercosis due to *T. solium* were detected in 2004. One case was a pig bought and raised for home consumption on a farm located near Coimbra (Unpublished data, Correia da Costa, 2016). The second case, a pig of the Bisaro breed (traditionally raised outdoors), was detected and confirmed at an abattoir in Vinhais (northern Portugal) [43, 44]. More recently, and according to official data from 2008 to 2015, no cases of *T. solium* cysticercosis were detected in Portugal (unpublished data, DGAV, 2016).

#### Bovine cysticercosis

**Fig. 8:**
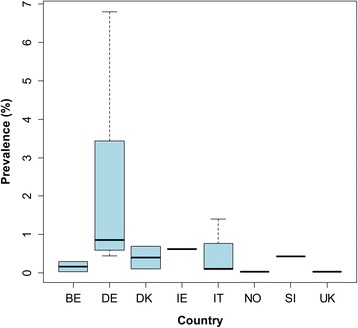
Prevalence of bovine cysticercosis based on routine meat inspection detected in western Europe before 1990. Prevalence estimates are from individual studies, and not the estimated prevalence for the entire country. *Abbreviations*: BE, Belgium; DE, Germany; DK, Denmark; IE, Ireland; IT, Italy; NO, Norway; SI, Slovenia; UK, United Kingdom

**Fig. 9:**
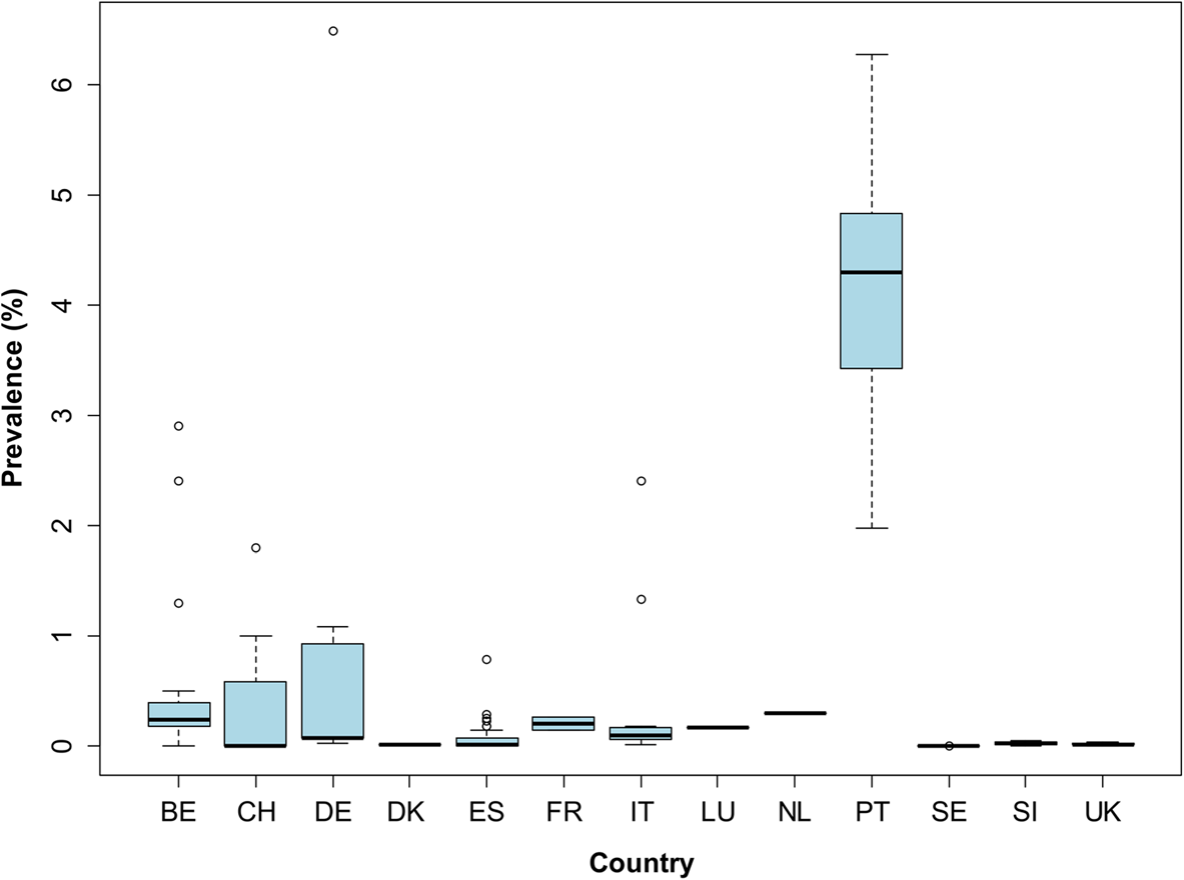
Prevalence of bovine cysticercosis based on routine meat inspection detected in western Europe after 1990. Prevalence estimates are from separate local studies. Data for Portugal correspond to the Autonomous Region of Madeira. Prevalences higher than 6.5%, which correspond to prevalences up to 7.82% detected in Madeira (2010), are not presented in the figure. *Abbreviations*: BE, Belgium; CH, Switzerland; DE, Germany; DK, Denmark; ES, Spain; FR, France; IT, Italy; LU, Luxembourg; NL, The Netherlands; PT, Portugal; SE, Sweden; SI, Slovenia; UK, United Kingdom

In our review we identified 85 sources providing unique information (prevalence or number of cases) from all (18) countries. Prevalence data or number of cases were mainly based on routine meat inspection (Regulation (EC) No 854/2004) [45]. Prevalence data of bovine cysticercosis was identified in fifteen out of the eighteen countries (Figs. 8, 9). For few countries and specific years, we retrieved the number of positive cases detected per year (prevalence data was not available) (Additional file 5: Table S13). Prevalence data based on more sensitive methods than routine meat inspection (i.e. serology or a more detailed meat inspection) [46, 47] were only available for six countries (Additional file 5: Table S14). In Iceland it has been never detected. However, it should be noted that incisions in the heart and masseter muscles are not routinely performed as part of meat inspection in Iceland [48].

The majority of bovine cysticercosis cases identified were detected after 1990. Figures 8 and 9 show the reported prevalence detected at slaughter before 1990 and after 1990, respectively. Prevalence reported before 1990 ranged from 0.03% (Belgium in 1969–1989 and Norway in 1989) to 6.80% (Former German Democratic Republic in 1974–1989). After 1990, the prevalence ranged from 0% (some regions of Spain in 2009–2014, one abattoir in Belgium in 2003, the UK in 2006 and mainland Portugal during 2008–2015) up to 7.82% (Madeira, Portugal, in 2010). After 1990, 95% of the prevalence data reported were below 4.87% and 50% were below 0.07%. The highest prevalence was reported in Madeira (7.82%). Although no positive cases were found in the Portuguese Autonomous region of Azores, at least part of the cases detected in Madeira seemed to have acquired the infection in Azores [49].

For Ireland and Norway, only one prevalence record before 1990 was available: 0.62% in Ireland (1977–1980) [50] and 0.03% in Norway (1989) [51]. However, individual cases were reported in Norway after this date [52, 53]. For Finland, no prevalence data could be retrieved, but 2 cases were reported: one in 1996 and one in 2002 (Additional file 5: Table S13).

In some reports on bovine cysticercosis, information on the degree of infection was available. The percentage of heavily infected cases ranged from 0.59 to 6.06% in Austria (1998–2003), 0.49–1.61% in Belgium (2002–2013), 5.30–6.47% in Germany (2009–2012) and 6.29–12.68% in Madeira (2007–2013).

Prevalence data based on more sensitive methods (i.e. serology, detailed meat inspection or modelling) ranged from 0.54 to 38.4% (Additional file 5: Table S14). Data on the occurrence of bovine cysticercosis according to the age of the animal was available for four countries: the prevalence in calves and adult cattle ranged between 0 and 0.55% and 0.03–1.68%, respectively (Additional file 5: Table S15).

## Discussion

The aim of the current study was to collect epidemiological data on *T. saginata* and *T. solium* in human and animal hosts in western Europe. Human taeniosis cases were identified in two thirds of the countries included in the search. Overall, the number of sources providing data was limited and the annual number of taeniosis cases found equally low for most countries, except for the UK and Spain. However, estimates based on anthelmintic sales (e.g. niclosamide) [25, 26, 54–58] or detection of Taeniidae eggs in sewage [27], although approximate, suggest that the true number of taeniosis cases is far from negligible. Indeed, we assume a serious underestimation, due to the fact that taeniosis is not a notifiable disease, the perceived low health impact, and a possible low awareness among medical doctors about the potential presence of *T. solium* carriers [14, 59] with a high public health impact. We further hypothesize that, as a consequence, the diagnosis is often based exclusively on patient’s reporting shedding of proglottids without any laboratory confirmation. Our results also highlight that species differentiation is rarely performed for taeniosis cases, reflected by the high proportion of cases reporting “*Taenia* spp.” as the causative agent. Next to the reasons discussed previously related to the perceived low health impact of the disease, diagnostic limitations might play a role for those cases for whom stool examination was performed. Indeed, *Taenia* spp. eggs are morphologically identical, and while differentiation can be made based on the number of uterus branches of expelled proglottids, such material is not always available. Furthermore, stool examination by molecular methods is not often performed [60]. Overall, given the lack of species differentiation in addition to the overall assumed underestimation of cases, it is difficult to estimate the true number of taeniosis cases caused by either *T. saginata* or *T. solium* in western Europe.

*Taenia saginata* is responsible for continuous economic losses for the meat industry, due to the condemnation or freezing of affected carcasses [3, 61] as prescribed in the European Regulation (EC) No 854/2004 [45]. Carriers of *T. saginata* contribute to these financial losses by sustaining the parasite’s life-cycle. In our search, *T. saginata* taeniosis cases were identified in Austria, Belgium, Denmark, Finland, France, Germany, Italy, Netherlands, Portugal, Spain, Slovenia and the UK. The presence of bovine cysticercosis was reported in nearly all countries included in the search, at different prevalence levels. As most of the data on *T. saginata* in cattle retrieved were based on meat inspection, a hugely insensitive detection method (reported sensitivity of 15.6%; [62]), we assume an underestimation of cases [46, 63]. A few false positive cases could also be present, as other causes of macroscopic lesions (e.g. abscesses, *Sarcocystis* cysts) could be confused with calcified cysticerci by meat inspection [59, 64]. Thus, more sensitive diagnostic tools should be implemented and species differentiation should be done in case of doubt. Furthermore, data reporting should be improved. Austria, for example, used to report findings on *T. saginata* at slaughter (1998–2003) but at present any (unspecified) cysts found in cattle are reported under the term “echinococcosis” [65–71]. For some countries (e.g. Norway, Finland) only sporadic cases of bovine cysticercosis were reported, which could be due to the lack of good reporting systems, as well as to the low prevalence or even absence of the parasite, due to the lack of favourable conditions for its transmission in these areas (e.g. lack of raw meat consumption, or lack of environmental factors such as use of sewage sludge on pastures).

*Taenia solium* taeniosis cases were reported in Denmark, France, Italy, Portugal, Spain, Slovenia and the UK, but the diagnostic methods used for identifying the *Taenia* species were often not clearly described [72]. On the individual level, identification of *T. solium* taeniosis cases is extremely relevant as one tapeworm carrier, if not treated, can pose a significant health risk to both themselves and to people in contact, as ingestion of infective eggs can lead to cysticercosis [11]. From the available data, it was not clear whether any of the reported *T. solium* taeniosis cases could have been acquired within western Europe through consumption of infected pork. However, as we can assume that most *T. solium* taeniosis cases were imported, prevalence studies in risk groups, such as travellers and immigrants, would be recommended. Furthermore, the epidemiological situation of *T. solium* in pigs was found to be unclear for many countries in western Europe: only five countries reported porcine cysticercosis cases and they usually did not report the causative species (i.e. cysticerci could also be *T. hydatigena*). Moreover, current reporting systems are often not consistent. For instance, in Austria similar to the cattle data previously discussed, nowadays only unspecified cysts are being reported for pigs [65–71]. Given the public health impact of *T. solium*, and because cysts of different *Taenia* spp. may not be distinguishable in the early stages [9], molecular confirmation should be performed in suspected porcine cysticercosis cases and the reporting should be made at species level, as recommended by EFSA [73]. Only Portugal reported two cases of *T. solium* in pigs, confirmed by molecular methods, one pig being raised outdoors and another bought for home consumption) (Correia da Costa, pers. com., 2016) [43, 44] supporting the hypothesis that in some rural areas in western Europe, favourable conditions might still exist for *T. solium* transmission (e.g. outdoor pig farming and contact with faeces from tapeworm carriers). In theory, increasing immigration and travels, combined with increasing outdoor pig farming (e.g. organic pig farming) may contribute to a future re-establishment of *T. solium* local transmission in many areas [9, 10] and we may expect there to be a rise in porcine cysticercosis cases in western Europe in the near future [9, 10].

Humans can act as dead-end host for *T. solium*, upon ingestion of eggs shed by a *T. solium* tapeworm carrier. The burden of human cysticercosis, especially in cases of NCC, is massive and it is believed to be the food-borne parasitic infection incurring the largest number of disability adjusted life years globally [74]. We found human cysticercosis cases in all western European countries included in the search, except Iceland. In some countries (e.g. Belgium, Finland, Ireland, Luxembourg, Norway, Sweden and Switzerland) included in our search, a cysticercosis case seemed to be a rare finding, whereas in countries like France, and especially the most southern countries in our search (Spain and Portugal), cases were more frequently observed. Based on the available epidemiological information, it was apparent that most human cysticercosis cases diagnosed in western Europe were linked to immigration or travel to endemic countries. The absolute number of cases in immigrants appear to have increased in recent years, with a large number of cases originating from Latin America and the Caribbean, possibly due to a rapid increase in immigration from this area towards Europe, mostly to the southern European countries, around the transition from the 20th to the twenty-first century [75]. Immigration from Africa has increased throughout the last decade and is expected to increase further [76]; we might therefore observe a rise in imported cases from African countries in the coming years. In addition, some cysticercosis cases originated from eastern Europe where favourable conditions for local *T. solium* transmission also seem to exist [8, 10]. Increased mobility, possibly associated with the introduction of the Schengen zone [9], could thus also result in a rise of imported cases from that region. In our review, we identified few human cysticercosis cases suspected to be autochthonously acquired. However, the exact place and time of infection and whether local transmission from an imported *T. solium* tapeworm carrier might have occurred could not be determined from the available data. Overall, although false positive cases of cysticercosis are possible in serological tests due to cross-reactions [77], the number of NCC cases identified in our search is probably lower than the actual number, as some NCC cases might not exhibit symptoms [78], the serological reference test exhibits a low sensitivity in case of single viable or calcified lesions [79], and clinicians in these non-endemic areas lack experience with the disease and therefore might not recognize it [59].

## Conclusions

The fact that both taeniosis and human cysticercosis are mainly non-notifiable diseases implies the absence of systematic data collection and reporting, leading to fragmented data. Overall, due to the economic impact of *T. saginata* and the potential impact on public health of *T. solium*, the improved detection and reporting of human taeniosis cases is extremely relevant for control and surveillance purposes. By maintaining the parasite life-cycle, *T. saginata* tapeworm carriers contribute to continuous economic losses in the meat sector. Furthermore, despite the low health impact, acquiring *T. saginata* taeniosis should not be acceptable from a food safety perspective. The existence of *T. solium* tapeworm carriers, combined with the presence of suspected autochthonous cases of human cysticercosis as well as the lack of confirmation of porcine cysticercosis cases in most countries, deserves further attention. We might see a rise in imported human cysticercosis in the near future due to increased migration from endemic countries. Species identification of taeniosis cases should be encouraged and epidemiological investigations carried out to detect whether local transmission of *T. solium* may occur. Furthermore, suspected cases of *T. solium* in pigs should be confirmed by molecular methods. Both taeniosis and human cysticercosis should be notifiable and surveillance and reporting in animals should be improved.

## Additional files

Additional file 1: Table S1. PRISMA 2009 Checklist. (DOC 64 kb)

Additional file 2: Country sheets template. (DOCX 47 kb)

Additional file 3: Table S2. List of references included in the review retrieved through online international databases. (XLSX 30 kb)

Additional file 4: Table S3. List of references included in the review made available through local sources. (XLSX 39 kb)

Additional file 5: Table S4. Identified taeniosis cases in case reports in western Europe (1990–2015). **Table S5.** Aggregated taeniosis cases reported in authorities’ reports, epidemiological bulletins, and national registries in western Europe (1990–2015). **Table S6.** Aggregated taeniosis cases reported at hospital/laboratory level in western Europe (1990–2015). **Table S7.** Taeniosis prevalence data reported in epidemiological studies (1990–2015). **Table S8.** Taeniosis estimates published in western Europe (1990–2015). **Table S9.** Identified human cysticercosis cases in case reports in western Europe (1990–2015). **Table S10.** Aggregated human cysticercosis cases identified in registries and reports in western Europe (1990–2015). **Table S11.** Aggregated human cysticercosis cases reported at hospital/laboratory level in western Europe (1990–2015). **Table S12.** Porcine cysticercosis cases and prevalence reported in western Europe (1990–2015) based on meat inspection. **Table S13.** Bovine cysticercosis cases reported (when prevalence not available) in western Europe (1990–2015) based on meat inspection. **Table S14.** Bovine cysticercosis prevalence detected in western Europe (1990–2015) by more sensitive methods than routine meat inspection. **Table S15.** Bovine cysticercosis prevalence data per age reported in western Europe (1990–2015) based on routine meat inspection. (DOCX 99 kb)

## Abbreviations

CYSTINET: European Network on Taeniosis/Cysticercosis
EFSA: European Food Safety Authority
GDP/GNI: Gross domestic product/Gross national income
ICD: International Classification of Diseases
NCC: Neurocysticercosis
OIE: World Organisation for Animal Health/Office International des Epizooties
