# Supplementary material for: Epidemiology of taeniosis/cysticercosis in Europe, a systematic review: Western Europe
Source: Parasit Vectors. 2017 Jul 21;10:349. doi: 10.1186/s13071-017-2280-8 (PMC5521153; doi:10.1186/s13071-017-2280-8)
Supplement: Supplementary file 5 — Identified taeniosis cases in case reports in western Europe (1990–2015). Table S5. Aggregated taeniosis cases reported in authorities’ reports, epidemiological bulletins, and national registries in western Europe (1990–2015). Table S6. Aggregated taeniosis cases reported at hospital/laboratory level in western Europe (1990–2015). Table S7. Taeniosis prevalence data reported in epidemiological studies (1990–2015). Table S8. Taeniosis estimates published in western Europe (1990–2015). Table S9. Identified human cysticercosis cases in case reports in western Europe (1990–2015). Table S10. Aggregated human cysticercosis cases identified in registries and reports in western Europe (1990–2015). Table S11. Aggregated human cysticercosis cases reported at hospital/laboratory level in western Europe (1990–2015). Table S12. Porcine cysticercosis cases and prevalence reported in western Europe (1990–2015) based on meat inspection. Table S13. Bovine cysticercosis cases reported (when prevalence not available) in western Europe (1990–2015) based on meat inspection. Table S14. Bovine cysticercosis prevalence detected in western Europe (1990–2015) by more sensitive methods than routine meat inspection. Table S15. Bovine cysticercosis prevalence data per age reported in western Europe (1990–2015) based on routine meat inspection. (DOCX 99 kb) [file 13071_2017_2280_MOESM5_ESM.docx]

**Additional file 5: Table S4.** Identified taeniosis cases in case reports in western Europe (1990-2015).

| **Country of diagnosis/report** | **Age at diagnosis (years)** | **Gender** | **Country of origin** | ***Taenia* species reported as** | **Possible risk factors reported** | **Reference** |
| --- | --- | --- | --- | --- | --- | --- |
| Belgium | 40 | M | Not available | *Taenia* spp. | Not available | Van Tiggelen and Danse (1998) |
| Belgium | 61 | M | Belgium | *Taenia* spp.  (suspected *T.* *saginata)* | Consumed raw beef meat | Vuylsteke et al. (2004) |
| France | 56 | M | France | *T. saginata* | Not available | Beaujot et al. (2015) |
| France | 19 | M | France | *T. saginata* | Not available | Duran et al. (1992) |
| France | 55 | F | Not available | *T. solium* (putative) | Consumed raw pork meat and/or offal | Halfon et al. (2014) |
| France | 66 | M | France | *T. saginata* | Consumed raw meat | Mercky et al. (2014) |
| Germany | 90 | M | Not available | *T. saginata* | Not available | Hoffman and Jung (2004) |
| Italy | 6 | F | Italy | *T. saginata* | Not available | Di Pietro et al. (1996) |
| Italy | Child | M | Italy | *T. saginata* | Not available | Dutto et al. (2009) |
| Italy | 75 | M | Italy | *T. saginata* | Consumed raw meat | Martines et al. (2006) |
| Italy | 26 | M | Italy | *T. solium* | Farmer | Minciullo et al. (2009) |
| Italy | 48 | M | Italy | *T. saginata* | Not available | Paolantonio et al. (2006) |
| Italy | 81 | M | Italy | *Taenia* spp. | Not available | Pezzoli et al. (2015) |
| Netherlands | 29 | M | Morocco | *T. saginata* | Not available | Van Beurden et al. (2008) |
| Netherlands | 39 | F | Not available | *T. saginata* | Not available | Van Roermund and Klaase (2003) |
| Portugal | 70-79 | F | Not available | *Taenia* spp. | Not available | Faria et al. (2015) |
| Spain | 17 | M | Spain | *Taenia* spp. | Came from rural and agricultural area | Arenas Abad et al. (1992) |
| Spain | 44 | M | Not available | *Taenia* spp. | Not available | Carretero et al. (2010) |
| Spain | 47 | F | Not available | *Taenia* spp. | Not available | Carretero et al. (2010) |
| Spain | 19 | F | Spain | *T. solium* | Consumed raw pork meat | Fernández-Aranda et al. (2001) |
| Spain | 33 | M | Spain | *T. saginata* | Lived temporarily in Africa | Lopez-Caleya et al. (2015) |
| Spain | 51 | F | Spain | *Taenia* spp. | No clear epidemiological history of risk | Villafruela et al. (2009) |

**Additional file 5: Table S5.** Aggregated taeniosis cases reported in authorities’ reports, epidemiological bulletins, and national registries in western Europe (1990-2015).

| **Country of diagnosis/report** | **Year** | **Total number of cases reported** | | | **Level of data collection** | **Details** | **Reference** |
| --- | --- | --- | --- | --- | --- | --- | --- |
|  |  | ***Taenia* spp*.*** | ***T. saginata*** | ***T. solium*** |  |  |  |
| Denmark | 2012-2014 | 78 | Not determined | Not determined | National | Not available | National inpatient diagnosis register (pers. com., 2015) |
| Netherlands | 1986-1990 | 16 | Not determined | Not determined | Not available | Number of hospitalisations with taeniosis as primary diagnosis (ICD codes) | Treurniet (1993) |
| Portugal | 2000-2013 | 8 | 13 | 8 | National (excluding Autonomous regions of Madeira and Azores) | Cases with taeniosis diagnosis at hospital discharge (GDH, Grupos diagnósticos Homogéneos), Portuguese ICD-9-CM reporting system | Vilhena (pers. com., 2017) |
| Slovenia | 1996-2014 | 212 | 25 | 5 | National | Not available | NIJZ (1998-2003); NIJZ (2005a); NIJZ (2005b); NIJZ (2006-2015) |
| Spain | 1992-2008 | 429 | 429 | 8 | National | Cases reported voluntarily by laboratories through the microbiological information system (SIM) | SIM, cited in Boletín Salud Pública Navarra (2002); SIM, cited in: Fos Claver et al. (2000);  SIM (NA) |
| UK | 2000-2014 | 1312 | Not determined | 2 | National | Laboratory confirmed | DEFRA (2002, 2003, 2004a, 2004b, 2005, 2006, 2007, 2008, 2013, 2015a, 2015b) |

**Additional file 5: Table S6.** Aggregated taeniosis cases reported at hospital/laboratory level in western Europe (1990-2015).

| **Country of diagnosis/report** | **Year** | **Total number of cases** | | | **Travel/migration history** | **Hospital/Lab** | **Reference** |
| --- | --- | --- | --- | --- | --- | --- | --- |
|  |  | ***Taenia* spp*.*** | ***T. saginata*** | ***T. solium*** |  |  |  |
| Denmark | 1998-2013 | 9 | 5 | 1 | Not available | Department of Clinical Microbiology, Aalborg University Hospital | Aalborg University Hospital (pers. com., 2016) |
|  | 2005-2015 | 26 | Not determined | Not determined | Not available | Statens Serum Institut data | Stensvold (pers. com., 2017) |
| France | Not determined |  | 40 |  | Not available | Not available | Bouree (1991) |
|  | 1988-1998 |  |  | 2 | French, Cape-Verde, Madagascar. All had been in endemic areas (Africa, South-America) | Laboratoires de parasitologie de Marseille et Nice (Region of Provence-Alpes-Côte d'Azur) | Rousseau et al. (1999) |
| Italy | 2006-2008 | 4  (It includes cases of *T. saginata* and *T. solium*) | Not determined | Not determined | 3 Italians with no history of travel, 1 non-Italian | Catholic University Hospital, Rome - Retrospective study | Masucci et al. (2011) |
| Portugal | 2000-2004 | 37 | Not determined | Not determined | Residents of Madeira | Laboratory of the Madeira Regional Health Service Retrospective study | Afonso (2008) |
| Slovenia | 1993-2015 | 26 | Not determined | Not determined | Not available | Laboratory for Parasitology in Slovenia, Institute of Microbiology and Immunology, Faculty of Medicine, University of Ljubljana | Šoba (pers. com., 2015) |
| Spain | 1984-1994 |  | 18 | 3 | Immigrants | Unidad de Medicina de la Inmigración y Tropical, Hospital de Mataró - Retrospective study in African immigrants | Roca et al. (2002) |
|  | 1989-1999 |  | 2 | 1 | Immigrants | Tropical Medicine Unit (TMU) of the Ramón y Cajal Hospital in Madrid - Retrospective study in immigrants from tropical, subtropical areas and eastern Europe | López-Vélez et al. (2003) |
| UK | 1998-2002 |  |  | 1 | Reported as imported case | Addenbrooke’s hospital in Cambridge -Review of imported infections | Roberts and Lever (2003) |

**Additional file 5: Table S7.** Taeniosis prevalence data reported in epidemiological studies (1990-2015).

| **Country of diagnosis/report** | **Year** | **Prevalence (%)** | ***Taenia* species reported as** | **Travel/migration history** | **Level of data collection** | **Reference** |
| --- | --- | --- | --- | --- | --- | --- |
| Austria | 1990-2000 | 0.07 | *T. saginata* | NA | Federal public health laboratory (FPHL) in Innsbruck | Tomaso et al. (2001) |
| France | 1997 | 0.10 | *T. saginata* | NA | Prospective survey of adults called to serve in the army catering | Hernandez et al. (1997) |
| Germany | 1970-1986 | 0.05-0.09 | *Taenia spp.* | NA | Halle District | Lorenz (1992) |
| Italy | 2007-2009 | 0.27 | *Taenia spp.* | Italians and non-EU | Cà Granda IRCCS Foundation, Ospedale Maggiore Policlinico of Milan | Grande et al. (2011) |
|  | 2008-2009 | 0.19 | *Taenia spp.* | Immigrants | Four institutions, Naples | Gualdieri et al. (2011) |
|  | 2009 | 0.20 | *T. saginata* | Italians | Microbiological laboratory, Reggio Emilia | Guidetti et al. (2010) |
| Portugal | 2005-2006 | 0.26 | *Taenia spp.* | Residents of Madeira | Hospital, Madeira | Afonso (2008) |

**Additional file 5: Table S8.** Taeniosis estimates published in western Europe (1990-2015).

| **Country** | **Year** | **Number of cases annually** | **Prevalence (%)** | ***Taenia* species reported as** | **Level of data collection** | **Based on** | **Reference** |
| --- | --- | --- | --- | --- | --- | --- | --- |
| Belgium | 1980-1989 |  | 0.35-0.46 | *Taenia spp.* | National | Sales of niclosamide | Geerts (1992) |
|  | 2013 | 11350 |  | *Taenia spp.* | National | Sales of niclosamide | De Keulenaer (2013) |
| Denmark | 1986 |  | 0.02  (17:100000) | *Taenia spp.* | National | Sales of niclosamide | Ilsøe et al. (1990) |
| France | 05/1987-01/1989 |  | 1.5-2.7 | *T. saginata* | Regional (Caen urban area) | Quantification of Taeniidae eggs content in sludge | Barbier et al. (1990) |
|  | 1999-2000 | 64495 |  | *T. saginata* | National | Sales of niclosamide | InVS (2003) |
| Germany | 1976-1989 |  | 0.33-0.67 | NA | Regional (former East Germany) | Anthelmintics sales | Möbius (1993) |
|  | 1977-1986 |  | 0.31-0.41 | *Taenia spp.* | Regional (Halle District) | Anthelmintics sales | Lorenz (1992) |
| Italy | NA |  | 0.02-0.04 | NA | NA | Sales of specific antiparasitic drugs in man | Battelli (1999), cited in: Health SCoVMrtPH (2000) |

**Additional file 5: Table S9.** Identified human cysticercosis cases in case reports in western Europe (1990-2015).

| **Risk factors** | **Country of origin or nationality** | | | | | | | |
| --- | --- | --- | --- | --- | --- | --- | --- | --- |
|  | **Africa** | **America: Caribbean, Central and South** | **Asia** | **Europe: eastern** | **Europe: western** | **Not available^[[1]](#footnote-1)^** | **Total** | **%** |
| **Immigrant** | 35 | 77 | 39 | 15 |  | 2 | **168** | **61.1** |
| **Travelled/stayed in endemic region (eastern Europe, Africa, America, Asia)** |  |  |  |  | 24 | 33^[[2]](#footnote-2)^ | **57** | **20.7** |
| **No history of travels to endemic areas or immigration** |  |  |  |  | 13 | 1 | **14** | **5.1** |
| **Not available** |  |  |  |  | 13 | 23 | **36** | **13.1** |
| **Total general** | **35** | **77** | **39** | **15** | **50** | **59** | **275** | **100** |

**Additional file 5: Table S10.** Aggregated human cysticercosis cases identified in registries and reports in western Europe (1990-2015).

| **Country of diagnosis/report** | **Number of cases** | **Year** | **Data source** | **Details** | **Reference** |
| --- | --- | --- | --- | --- | --- |
| Denmark | 32 | 2012-2014 | National registry | Not available | National inpatient diagnosis register (pers. com., 2015) |
| Iceland | 0 | 2013-2014 | Governmental report | Not available | Embætti landlæknis (2015) |
| Italy | 540 | 2001-2010 | Number of hospitalisations for cysticercosis, Italian ICD-9-CM reporting system | Range of 40 and 53 hospitalisations per year  276 Italian citizens, 132 Latin America (51 Ecuador, 35 Peruvians, 17 Bolivians), 40 Africa, 78 Asia (64 India), 14 other from Europe. | Ministero della Salute, Istituto Nazionale di Statistica, cited in Zammarchi et al. (2013) |
| Netherlands | 24 | 1986-1990 | Number of hospitalisations with cysticercosis as primary diagnosis following ICD codes | Not available | Treurniet (1993) |
| Portugal | 1120 | 1993-2004 | Number of hospitalised cysticercosis cases from all Portuguese hospitals (Ministry of Health’s data base ‘Grupos de Diagnóstico Homogéneos’) | Larger cities: many were immigrants  Northern part: evidence of autochthonous cases.  14.7% were <24 years old | Vilhena et al. (2007) |
|  | 357 | 2006-2013 | Number of hospitalised NCC cases (national database on National Health Service hospital episodes; excluding Madeira and Azores Islands), Portuguese ICD-9-CM reporting System. | Mean of 45 cases per year.  In Northern Region cases tended to be older than in Lisboa and Vale do Tejo Region. NCC most frequent in 25-34 and >75 year-old | Vilhena et al. (2015) |
| Spain | 1702 | 1997-2014 | Number of hospitalised patients with diagnosis of cysticercosis at hospital discharge (CMBD-H^[[3]](#footnote-3)^), Spanish ICD-9-CM reporting system | Range of 45 and 169 hospitalisations per year | MSSSI. Instituto de Información Sanitaria. CMBD-H (2016) |

**Additional file 5: Table S11.** Aggregated human cysticercosis cases reported at hospital/laboratory level in western Europe (1990-2015).

| **Country of diagnosis/report** | **Cases** | **Year** | **Hospital/Lab** | **Travel/migration history** | | | **Details** | **Reference** |
| --- | --- | --- | --- | --- | --- | --- | --- | --- |
|  |  |  |  | **Endemic countries** | **Western Europe** | **Unknown** |  |  |
| Austria | 1 | 1996-2000 | ** |  |  | 1 | Not available | Overbosch et al. (2002) |
|  | 15 | 2004-2014 | Department of Neurology; Medical Univ. Innsbruck and Department of Parasitology, Medical University Vienna | 4 |  | 11 | 4 cases came from Innsbruck, all of them immigrants: two from the Balkan countries, one Tibetan and one Brazilian | Schmutzhard and Auer (pers. com., 2017) |
| Denmark | 10 | 2005-2015 | Statens Serum Institut data |  |  | 10 | Not available | Stensvold (pers. com., 2017) |
| France | 5 | 1997^[[4]](#footnote-4)^ |  |  |  | 5 | Not available | Dumas et al. (1997) |
|  | 93 | 1978-1988 | Main parasitology laboratories of the mainland France | 64 | 18 (mainly Iberian peninsula) | 11 | Reported place of infection: 40 Madagascar, 18 Europe (mainly Iberian peninsula), 9 Asia, 9 South/Central-America, 6 Africa | Lortholary et al. (1990) |
|  | 29 | 1988-1998 | Parasitology laboratories of Marseille and Nice and hospitals and clinics of the region (Provence-Alpes-Côte d'Azur) | 29 |  |  | French, Cape-Verde, Madagascar. All patients had lived or traveled in endemic areas (mainly equatorial and tropical Africa, but also in North Africa, Madagascar and South America) | Rousseau et al. (1999) |
|  | 8 | 1996-2000 | ** |  |  | 8 | Not available | Overbosch et al. (2002) |
| Germany | 6 | 1996-2000 | ** |  |  | 6 | Not available | Overbosch et al. (2002) |
| Italy | 20 | 1991-1994 | Laboratorio di Parassitologie, Instituto Superiore di Sanita, Roma | 3 | 17 (Italy) |  | Three had a prolonged stay in endemic countries (Latin-America). Case history showed that 17 probably acquired the infection in Italy | Tamburrini et al. (1995) |
|  | 11* | 2001-2010 | Center for Immigrant and Foreign Adopted Children, Meyer Hospital, Florence Italy | 11 |  |  | Immigrant and foreign adopted children: 6 Latin-American, 3 Ethiopian children, one unspecified African child and one Indian child | Zammarchi et al. (2011) |
|  | 59 | 2001-2014 | Istituto Superiore di Sanità | 59 |  |  | All had spent years in endemic countries | Gómez-Morales et al. (2015) |
| Netherlands | 107 | 1996-2004 | National Institute for Public Health and Environment (RIVM) |  |  | 107 | Not available | Kortbeek (pers. com., 2015) |
|  | 26 | 2004-2013 | National Institute for Public Health and Environment (RIVM) |  |  | 26 | Not available | Kortbeek (pers. com., 2015) |
|  | 14 | 1996-2000 | ** |  |  | 14 | Not available | Overbosch et al. (2002) |
| Norway | 1 | 1996-2000 | ** |  |  | 1 | Not available | Overbosch et al. (2002) |
|  | 5 | 1994^[[5]](#footnote-5)^ |  | 4 |  | 1 | 1 case originally from India, 1 from Chile, and 1 from Vietnam; the 2 other cases were Norwegian, but one had lived in Africa for several years | Dietrichs et al. (1994) |
| Portugal | 348 | 1983-1992 | Hospital Geral de Santo António, Oporto |  |  | 348 | Not available | Monteiro (1995b) |
|  | 35 | 1987-1992 | Santa Maria Hospital, Lisbon | 17 |  | 18 | 17 were of African origin: natives or residents of former Portuguese colonies | Morgado et al. (1994) |
|  | 14 | 1996-2003 | General Hospital at Lisbon Metropolitan Area | 13 |  | 1 | 93% of cases (13) from African origin | Ferreira et al. (2006) |
|  | 53 | 2003-2013 | Santa Maria Hospital, Lisbon | 53^[[6]](#footnote-6)^ |  |  | Mostly imported from Portuguese-speaking African countries | Valadas et al. (2015) |
|  | 11 | 1989-2000 | Hospital de S. Francisco Xavier, Lisbon | 10 | 1 (Portugal) |  | Likely place of infection reported: 10 in Africa (7 Cape Verde, 2 Guiné-Bissau, S. Tomé e Príncipe), 1 in Portugal | Flores et al. (2001) |
|  | 15 | 2010-2015 | Centro Hospitalar Lisboa Central, Lisbon | 15^[[7]](#footnote-7)^ |  |  | Most from Cape Verde | Januário et al. (2015) |
| Slovenia | 5 | 1991-2000 | Laboratory for Parasitology, Institute of Microbiology and Immunology, Faculty of Medicine, Ljubljana |  |  | 5 | According to authors: probably infected while visiting relatives in the countries of former Yugoslavia | Rakuša (2000) |
|  | 2 | 2013-2015 |  |  |  | 2 | Not available | Šoba (pers. com., 2015) |
| Spain | 18 | 1960-1996 | Fundacion Jimenez Diaz, Madrid |  |  | 18 | 16 Spanish (10 from rural areas and they all were seen before 1988) | Bello Martínez et al. (1997) |
|  | 13 | 1980-1989 | Hospital General Universitario Gregorio Marañón, Madrid | 2 |  | 11 | 11 Spanish-born, 2 foreign immigrants (Latin-America) | Esquivel et al. (2005) |
|  | 20 | 1990-2002 | Hospital General Universitario Gregorio Marañón, Madrid | 17 |  | 3 | 3 Spanish (migrated from rural endemic areas in the past) and 17 immigrants (16 Latin-Americans, 1 African) | Esquivel et al. (2005) |
|  | 10 | 1986-1991 | Hospital Nuestra Señora del Cristal, Ourense | 1 |  | 9 | One was originally from Brazil | Fernández-Rodríguez et al. (1991) |
|  | 31 | 1989-2008 | Tropical Medicine Unit of the Ramón y Cajal Hospital, Madrid | 31 |  |  | Review of 2198 immigrants: Cysticercosis in 3 Sub-Saharan Africans, 28 Latin-Americans | Monge-Maillo et al. (2009) |
|  | 19 | 1990-2008 | University Hospital Puerta De Hierro Majadahonda | 13 |  | 6 | 13 Latin-Americans | Minguito Parra et al. (2009) |
|  | 25 | 1992-2002 | Tropical Medicine Unit of the University Hospital Clinic; Parc Taulí | 23 | 2 |  | 2 native non-travellers and 23 imported (17 immigrants and 6 European travellers) | Roca et al. (2003) |
|  | 38 | 1996-2006 | La Paz University Hospital | 15 |  | 23 | More common among immmigrants from Latin America (15 were from Ecuador) | Fernandez-Dominguez et al. (2007) |
|  | 35^[[8]](#footnote-8)^ | 1996-2009 | Hospital Vega del Río Segura, Cieza,  Hospital Universitario Virgen de la Arrixaca | 35 |  |  | Latin-Americans | Ruiz et al. (2011) |
|  | 23*** | 1997-2005 | El Hospital Virgen de la Arrixaca | 20 |  | 3 | 20 Latin-Americans | Más-Sesé, et al. (2008) |
|  | 10 | 2000-2006 | Hospital Universitario Central de Asturias | 6 |  | 4 | 4 were Spanish and 6 were immigrants (4 from Ecuador and 2 from Brasil) | Rodríguez Guardado et al. (2007) |
|  | 10 | 2001-2010 | Hospital General Universitario de Elche | 10 |  |  | Review of 1071 immigrant patients: 10 cases were Latin-Americans | Ramos et al. (2011) |
|  | 5 | NA | Hospital Nuestra Señora Virgen del Puerto |  |  | 5 | Study on aetiology of epilepsy in a rural health care area | Jiménez Jiménez et al. (1990) |
|  | 16 | 1998-2002 | NA | 15 |  | 1 | 15 Latin-Americans, 1 unknowm | Lobo et al. (2003) |
|  | 9 | 1996-2000 | ** |  |  | 9 | Not available | Overbosch et al. (2002) |
| Sweden | 9 | 1999 - 2004 | Smittskyddsinstitutet Solna |  |  | 9 | Among these there are Swedish people who have been abroad or originally from South America, Africa or Asia, but background or nationality is not known for all cases | Puschmann et al. (2006) |
| Switzerland | 1 | 1996-2000 | ** |  |  | 1 | Not available | Overbosch et al. (2002) |
|  | 6 | 2000-2010 | Geneva University  Hospitals | 4 |  | 2 | 2 were Swiss citizends living in Geneva, while the others were from endemic zones | Cruz and Burkhard (2010) |
| UK | 2 | 1996-2000 | ** |  |  | 2 | Not available | Overbosch et al. (2002) |
|  | 2 | 1998-2002 | Addenbrooke’s hospital in Cambridge | 2 |  |  | Imported cases | Roberts and Lever (2003) |

** Overbosch et al. (2002): Information gathered via Public Health Laboratories, Institutes of Tropical Diseases (survey) and doctors (questionnaires).

**Additional file 5: Table S12.** Porcine cysticercosis cases and prevalence reported in western Europe (1990-2015) based on meat inspection.

| **Country of diagnosis/report** | **Prevalence range (%)** | **Cases** | **Year** | **Level of data collection** | **Reported as** | **References** |
| --- | --- | --- | --- | --- | --- | --- |
| Austria |  | 0-40 cases/year | 1998-2002 | National | *“C. cellulosae”* | Bundeskanzleramt (1998); Bundesministerium für soziale Sicherheit und Generationen (1999, 2000, 2001); Bundesministerium für Gesundheit und Frauen (2002) |
|  |  |  | 2007 | National | Porcine cysticercosis | Dorny et al. (2010) |
| Belgium |  | 0 | 2002-2013 | National | Porcine cysticercosis | EFSA (2004, 2005, 2006, 2007, 2008, 2009, 2010, 2011, 2012, 2013)  Dorny et al. (2010) |
| Denmark |  | 0 | 2007 | National | Porcine cysticercosis | Dorny et al. (2010) |
| Finland |  | 0 |  | National | Porcine cysticercosis | EVIRA (2015) |
| Germany |  | 0 | 2007 | National | Porcine cysticercosis | Dorny et al. (2010) |
|  | 0-0.0023 | 16/50289738 (2009);  14/53208257 (2010);  1278/55078995 (2011);  0/53900913 (2012) | 2009-2012 | National | *“C. cellulosae”* or “*C.* *tenuicollis”* (no distinction between them) | Nagel-Kohl (2014) |
| Ireland |  | 0 | Not specified | National | *“C. cellulosae”* | FSAI (2008) |
| Italy |  | 0 | 2007 | National | Porcine cysticercosis | Dorny et al. (2010) |
| Luxembourg |  | 0 | Not available | National | Porcine cysticercosis | Dorny et al. (2010) |
| Netherlands |  | 0 | 2006 | National | Porcine cysticercosis | Dorny et al. (2010) |
| Portugal |  | 1 | 2004 | Farm | *T. solium* | Correia da Costa (pers. com., 2016) |
|  | 0.2326 | 1/430 | 2004 | Abattoir | *T. solium* | Freire (2005); Vieira-Pinto (2015) |
|  |  | 0 | 2005 | National | Porcine cysticercosis | Dorny et al. (2010) |
|  |  | 0 | 2008-2015 | National | *T. solium* | DGAV (pers. com., 2016)^[[9]](#footnote-9)^ |
| Slovenia | 0.0002 | 1/424634 | 2007 | National | Porcine cysticercosis (No species distinction) | UVHVVR (2015) |
|  | 0 | 0/384400 | 2008 | National | Porcine cysticercosis (No species distinction) | UVHVVR (2015) |
|  | 0 | 0/295491 | 2009 | National | Porcine cysticercosis (No species distinction) | UVHVVR (2015) |
|  | 0 | 0/291120 | 2010 | National | Porcine cysticercosis (No species distinction) | UVHVVR (2015) |
|  | 0 | 0/280266 | 2011 | National | Porcine cysticercosis (No species distinction) | UVHVVR (2015) |
|  | 0 | 0/252894 | 2012 | National | Porcine cysticercosis (No species distinction) | UVHVVR (2015) |
|  | 0 | 0/229066 | 2013 | National | Porcine cysticercosis (No species distinction) | UVHVVR (2015) |
|  | 0 | 0/241286 | 2014 | National | Porcine cysticercosis (No species distinction) | UVHVVR (2015) |
| Spain  (domestic pig) | 0-0.198 |  | 1999-2014 | Regional | Porcine cysticercosis (No species distinction) | Consejería de Sanidad de la Región de Murcia (pers. com., 2015); AECOSAN (2011); Consejería de Igualdad, Salud y Políticas Sociales, Junta de Andalucía (pers. com., 2015); Dirección General de Salud Pública, Conselleria de Sanitat de la Generalitat Valenciana (pers. com., 2015); D. G. de Salud Pública, Consejería de Sanidad, Junta de Castilla y León (pers. com., 2015); AECOSAN (pers. com., 2015); Consellería de Sanidade, Xunta de Galicia (pers. com., 2015); ASPCAT (pers. com., 2015); Dirección General de Salud Pública y Consumo del Gobierno de La Rioja (pers. com., 2015) |
|  | 0 | 0/ 483735 | 2002-2011 | Regional | *T. solium* | Sánchez Martínez (2013) |
| Spain  (home-slaughtering) | 0.16-0.43 |  | 2011-2013 | National | Porcine cysticercosis (No species distinction) | AECOSAN (pers. com., 2015) |
| Spain  (wild boar) | 0-0.19 |  | 2009-2013 | Regional | Porcine cysticercosis (No species distinction) | Consejería de Igualdad, Salud y Políticas Sociales, Junta de Andalucía (pers. Com., 2015); AECOSAN (pers. com., 2015) |
| Spain  (extensive breeding) | 0 | 0/689 | Not specified | Regional | *T. solium* | García Vallejo (1999) |
| Sweden | 0 |  | 2011-2014 | National | *T. solium* | EFSA (2015a); EFSA (2015b) |
| UK | 0 |  | NA | National | *T. solium* | DEFRA (2003) |

**Additional file 5: Table S13.** Bovine cysticercosis cases reported (when prevalence not available) in western Europe (1990-2015) based on meat inspection.

| **Country of diagnosis/report** | **Cases per year** | **Year** | **Level of data collection** | **Reference** |
| --- | --- | --- | --- | --- |
| Austria | Between  295-545 | 1998-2003 | National | Bundeskanzleramt (1998); Bundesministerium für soziale Sicherheit und Generationen (1999, 2000, 2001); Bundesministerium für Gesundheit und Frauen (2002, 2003) |
|  | 204 | 2007 | National | Dorny et al. (2010) |
| Belgium | 3336 | 2002 | National | EFSA (2004) |
| Finland | 1 | 1996 | National | Hallanvuo and Johansson (2010) |
|  | 1 | 2002 | National |  |
| Luxembourg | 125 | Not available | National | Dorny et al. (2010) |
| Netherlands | 557 | 2008 | National |  |
|  | 28 | 2012 | Regional (one farm) | RIVM (2013) |
| Norway | 2 herds | 2011 | Regional (northern Norway) | NVI (2012) |
| Switzerland | 43 | Not available | Regional (North-East Switzerland) | Van der Logt and Gottstein (2000) |
| UK | 565 | 2005 | Regional (Scotland, 65 abattoirs) | FSA Scotland (2008) |

**Additional file 5: Table S14.** Bovine cysticercosis prevalence detected in western Europe (1990-2015) by more sensitive methods than routine meat inspection.

| **Country of diagnosis/report** | **Prevalence range (%)** | **Cases/Sampled** | **Year** | **Level of data collection** | **Diagnostic** | **Reference** |
| --- | --- | --- | --- | --- | --- | --- |
| Belgium | 3.09 (0.9-5.0) | 36/1164 | 11/1997-06/1998 | National (20 export abattoirs) | AgELISA | Dorny et al. (2000) |
|  | 38.4 | 19194/500000 | yearly | National | Dissection, Ag +Ab ELISA + modelling | Jansen et al. (2015) |
|  | 9.5 |  | Not available | National | Detailed meat inspection | Geerts (1990) |
| Germany | 8.83 (1.61-33.3) | 134/1518 | 03/2007-01/2008 | Regional (districts of the federal state of Lower Saxony)^[[10]](#footnote-10)^ | AbELISA | Abuseir et al. (2010) |
| Portugal | 12.9 | 9/70 | 2013 | Regional (born and tested in Azores slaughtered in Madeira) | ELISA = Bovine Cysticercosis antibody (CYT Ab) ELISA kit® (GENTAUR) | Antunes (2014) |
| Slovenia | 30.2 | 19/63 | 2015 | Regional (one farm found positive by meat inspection) | Ag ELISA | Vergles-Rataj et al. (2015) |
| Spain | 1.11 (95% C.I.:0.76–1.75) | 727/2073 | Nov 2009 - Feb 2010 | Regional (Catalonia) | Ag ELISA (B158/B60 antigen ELISA) | Allepuz et al. (2012) |
|  | 0.54 (0.41-0.75) | 232/43289 | 1992-1998 | Regional (northern Spain) | Detailed meat inspection targeting cysticercosis | García-Castro (2003), cited in EFSA (2004b) |
| Switzerland | 4.5 | 49/1088 | 11/2008-10/2009 | Regional (3 EU-approved abattoirs) | Meat inspection + enhanced heart examination | Eichenberger et al. (2011) |
|  | 16.5 (95% C.I.:12.50–21.20)^[[11]](#footnote-11)^ |  |  | National (dairy cows slaughtered in Swiss abattoirs) | Multitesting (meat inspection + 4 serological tests) | Eichenberger et al. (2013) |

**Additional file 5: Table S15.** Bovine cysticercosis prevalence data per age reported in western Europe (1990-2015) based on routine meat inspection.

| **Country of diagnosis/report** | **Age** | **Prevalence (%)** | **Cases/sampled** | **Year** | **Level of data collection** | **Reference** |
| --- | --- | --- | --- | --- | --- | --- |
| Belgium | Calves (<1y.) | 0.00096 | 3/313115 | 2005 | National | EFSA (2005) |
|  | Adults (>2y.) | 0.46 | 2389/523795 |  |  |  |
| Italy | Calves | 0 | 0/472 | 1991 | 2 abattoirs and 4 cutting plants (Chiavari) | Poirè et al. (1994) |
|  | Steers | 1.68 | 29/1726 |  |  |  |
|  | Cows | 1.5 | 4/266 |  |  |  |
|  | Calves | 0.555 | 13/2342 | 1980-1985 | 1 abattoir (Piedmont) | Julini et al. (1993) |
|  | Steers | 1.472 | 446/30302 |  |  |  |
|  | Cows | 1.429 | 22/1540 |  |  |  |
| Netherlands | Veal calves | 0.002 | Not available | 2011 | National | NVWA (2013) |
|  | Adults | 0.3 | Not available |  |  |  |
| United Kingdom | Calves | 0.008 | 15/190493 | 2008-2011 | National | Hill et al. (2014) |
|  | Adults | 0.032 | 2674/8484371 |  |  |  |

# References

Aalborg University Hospital, Department of Clinical Microbiology. Denmark. 2016. Personal communication.

Abuseir S, Nagel-Kohl U, Probst D, Kuhne M, Epe C, Doherr MG, et al. Seroprevalence of *Taenia saginata* cysticercosis in the federal state of Lower Saxony in Germany. Berl Munch Tierarztl Wochenschr. 2010;123(9-10):392-6.

AECOSAN (Agencia Española de Seguridad Alimentaria y Nutrición). Informe anual del Sistema Nacional de Salud 2009. Ministerio de Sanidad, Política Social e Igualdad. Informes, Estudios e investigación 2011. 2011. https://www.msssi.gob.es/organizacion/sns/planCalidadSNS/pdf/equidad/informeAnual2009/InformeAnualSNS2009.pdf. Accessed Feb 2017.

AECOSAN (Agencia Española de Consumo, Seguridad Alimentaria y Nutrición). Ministerio de Sanidad, Servicios Sociales e Igualdad. Spain. 2015. Personal communication.

Afonso MBVdO. Prevalência de *Taenia saginata*/*Cysticercus bovis* na Região Autónoma da Madeira. Dissertação de mestrado em saúde pública veterinária. Universidade Técnica de Lisboa. 2008.

Allepuz A, Gabriël S, Dorny P, Napp S, Jansen F, Vilar MJ, et al. Comparison of bovine cysticercosis prevalence detected by antigen ELISA and visual inspection in the North East of Spain. Res Vet Sci. 2012;92(3):393-5.

Antunes GJ. Parasitismo muscular por *Sarcocystis* spp. e *Cysticercus bovis* (*Taenia saginata*) em bovinos da Região Autónoma dos Açores. Dissertação de mestrado integrado em medicina Veterinária. Universidade de Lisboa. 2014.

Arenas Abad A, Dominguez Gabas JL, Lorente Aznar T, Mateos Omiste J. A case of taeniasis in primary care. Aten Primaria. 1992;10(3):698-9.

ASPCAT (Agència de Salut Pública de Catalunya). Spain. 2015. Personal communication.

Barbier D, Perrine D, Duhamel C, Doublet R, Georges P. Parasitic hazard with sewage sludge applied to land. Appl Environ Microbiol. 1990;56(5):1420-2.

Battelli (1999), cited in SCoVMrtPH (Scientific Committee on Veterinary Measures relating to Public Health). Opinion of the Scientific Committee on Veterinary Measures relating to Public Health on The control of taeniosis/cysticercosis in man and animals (adopted on 27–28 September 2000). 2000. European Commission.

Beaujot J, Leteurtre E, Do Cao C, Beron A, Caiazzo R, Vantyghem MC. Potential role of parasitosis in tumorigenesis: case study of heart metastasis as the only presenting symptom of an ileal neuroendocrine tumor. Presse Med. 2015;44(1):102-6.

Bello Martinez E, de Gorgolas Hernandez-Mora M, Albisua Sanchez J, Ruiz Barnes P, Cuenca Estrella M, Sarasa Corral JL, et al. Neurocysticercosis in a tertiary hospital. New advances in the diagnosis and treatment. Rev Clin Esp. 1997;197(9):604-10.

Bouree P. Successful treatment of *Taenia saginata* and *Hymenolepis* *nana* by single oral dose of praziquantel. J Egypt Soc Parasitol. 1991;21(2):303-7.

Bundeskanzleramt. Veterinärjahresbericht 1998. Bundeskanzleramt Sektion VI, Veterinärverwaltung, A-1031 Wien.

Bundesministerium für soziale Sicherheit und Generationen. 1999. Veterinärjahresbericht 1999. Bundesministerium für soziale Sicherheit und Generationen, Sektion IX, Veterinärverwaltung, A-1031 Wien.

Bundesministerium für soziale Sicherheit und Generationen. 2000. Veterinärjahresbericht 2000. Bundesministerium für soziale Sicherheit und Generationen, Sektion IX, Veterinärverwaltung, A-1031 Wien.

Bundesministerium für soziale Sicherheit und Generationen. 2001. Veterinärjahresbericht 2001. Bundesministerium für soziale Sicherheit und Generationen, Sektion VII, Veterinärverwaltung, A-1031 Wien.

Bundesministerium für Gesundheit und Frauen. 2002. Veterinärjahresbericht 2002. Bundesministerium für Gesundheit und Frauen, Sektion IV, Veterinärverwaltung, 1030 Wien.

Bundesministerium für Gesundheit und Frauen. 2003. Veterinärjahresbericht 2003. Bundesministerium für Gesundheit und Frauen, Sektion IV, Veterinärverwaltung, 1030 Wien.

Carretero C, Borda A, Muñoz-Navas M. Face to face with teniasis. Clin Gastroenterol Hepatol. 2010;8(1):A36.

Consejería de Igualdad, Salud y Políticas Sociales. Junta de Andalucía. Spain. 2015. Personal communication.

Consejería de Sanidad de la Región de Murcia. Spain. 2015. Personal communication.

Consellería de Sanidade. Xunta de Galicia. Spain. 2015. Personal communication.

Correia da Costa JM. 2016. Personal communication.

Cruz CV, Burkhard PR. Neurocysticercosis (NCC): a not so rare parasitic infection in Geneva, Switzerland. European Journal of Neurology. 2010;17(Suppl. 3):576.

De Keulenaer. De epidemiologie van cysticercose en taeniose bij varkens, runderen en de mens in België. MSc Thesis. Ghent University. 2013.

DEFRA (Department for Environment, Food and Rural Affairs). Zoonoses Report United Kingdom 2000. 2002.

DEFRA (Department for Environment, Food and Rural Affairs). Zoonoses Report United Kingdom 2001. 2003.

DEFRA (Department for Environment, Food and Rural Affairs). Zoonoses Report United Kingdom 2002. 2004a.

DEFRA (Department for Environment, Food and Rural Affairs). Zoonoses Report United Kingdom 2003. 2004b.

DEFRA (Department for Environment, Food and Rural Affairs). Zoonoses Report United Kingdom 2004. 2005.

DEFRA (Department for Environment, Food and Rural Affairs). Zoonoses Report United Kingdom 2005. 2006.

DEFRA (Department for Environment, Food and Rural Affairs). Zoonoses Report United Kingdom 2006. 2007.

DEFRA (Department for Environment, Food and Rural Affairs). Zoonoses Report United Kingdom 2007. 2008.

DEFRA (Department for Environment, Food and Rural Affairs). Zoonoses Report UK 2012. 2013.

DEFRA (Department for Environment, Food and Rural Affairs). Zoonoses Report UK 2013. 2015a.

DEFRA (Department for Environment, Food and Rural Affairs). Zoonoses Summary Report UK 2014. 2015b.

DGAV (Direcção-Geral de Alimentação e Veterinária). Portugal. 2016. Personal communication.

Di Pietro A, Proverbio MR, Coletta M, Siani P, Zeoli L, Tammaro V. Parasitic arthritis: a case report. Pediatr Med Chir. 1996;18(2):211-2.

Dietrichs E, Tyssvang T, Aanonsen NO, Bakke SJ, Skullerud K. Tapeworms in the brain--a current problem in Norway?. Tidsskr Nor Laegeforen. 1994;114(26):3089-92.

Dirección General de Salud Pública y Consumo del Gobierno de La Rioja. Spain. 2015. Personal communication.

Dirección General de Salud Pública. Consejería de Sanidad. Junta de Castilla y León. Spain. 2015. Personal communication.

Dirección General de Salud Pública. Conselleria de Sanitat de la Generalitat Valenciana. Spain. 2015. Personal communication.

Dorny P, Vercammen F, Brandt J, Vansteenkiste W, Berkvens D, Geerts S. Sero-epidemiological study of *Taenia saginata* cysticercosis in Belgian cattle. Vet Parasitol. 2000;88:43–9.

Dorny P, Vallée I, Alban L, Boes J, Boireau P, Boué F, et al. Development of harmonised schemes for the monitoring and reporting of cysticercus in animals and foodstuffs in the European Union. EFSA Supporting Publication. 2010;7 1:EN-34, 30 pp.

Dumas JL, Visy JM, Belin C, Gaston A, Goldlust D, Dumas M. Parenchymal neurocysticercosis: follow-up and staging by MRI. Neuroradiology. 1997;39(1):12-8.

Duran A, Grassin F, Richardi G, Curtet M. Loeffer's syndrome: is *Taenia* responsible? Rev Pneumol Clin. 1992;48(6):279-81.

Dutto M, Giovanetti F, Pellegrino A. Teniasis in a child with finding of *Taenia saginata* proglottids in the school environment: a case report. Ann Ig. 2009;21(2):183-6.

EFSA (European Food Safety Authority). The Report referred to in Article 5 of Directive 92/117/EEC. Trends and sources of zoonoses and zoonotic agents in humans, foodstuffs, animals and feedingstuffs including information on foodborne outbreaks and antimicrobial resistance in zoonotic agents in 2004. 2004. Belgium.

EFSA (European Food Safety Authority). The Report referred to in Article 9 of Directive 2003/99/EC. Trends and sources of zoonoses and zoonotic agents in humans, foodstuffs, animals and feedingstuffs including information on foodborne outbreaks and antimicrobial resistance in zoonotic agents in 2005. 2005. Belgium.

EFSA (European Food Safety Authority). The Report referred to in Article 9 of Directive 2003/99/EC. Trends and sources of zoonoses and zoonotic agents in humans, foodstuffs, animals and feedingstuffs including information on foodborne outbreaks and antimicrobial resistance in zoonotic agents in 2006. 2006. Belgium.

EFSA (European Food Safety Authority). The Report referred to in Article 9 of Directive 2003/99/EC. Trends and sources of zoonoses and zoonotic agents in humans, foodstuffs, animals and feedingstuffs including information on foodborne outbreaks and antimicrobial resistance in zoonotic agents in 2007. 2007. Belgium.

EFSA (European Food Safety Authority). The Report referred to in Article 9 of Directive 2003/99/EC. Trends and sources of zoonoses and zoonotic agents in humans, foodstuffs, animals and feedingstuffs including information on foodborne outbreaks and antimicrobial resistance in zoonotic agents in 2008. 2008. Belgium.

EFSA (European Food Safety Authority). The Report referred to in Article 9 of Directive 2003/99/EC. Trends and sources of zoonoses and zoonotic agents in humans, foodstuffs, animals and feedingstuffs including information on foodborne outbreaks and antimicrobial resistance in zoonotic agents in 2009. 2009. Belgium.

EFSA (European Food Safety Authority). The Report referred to in Article 9 of Directive 2003/99/EC. Trends and sources of zoonoses and zoonotic agents in humans, foodstuffs, animals and feedingstuffs including information on foodborne outbreaks and antimicrobial resistance in zoonotic agents in 2010. 2010. Belgium.

EFSA (European Food Safety Authority). The Report referred to in Article 9 of Directive 2003/99/EC. Trends and sources of zoonoses and zoonotic agents in humans, foodstuffs, animals and feedingstuffs including information on foodborne outbreaks and antimicrobial resistance in zoonotic agents in 2011. 2011. Belgium.

EFSA (European Food Safety Authority). The Report referred to in Article 9 of Directive 2003/99/EC. Trends and sources of zoonoses and zoonotic agents in humans, foodstuffs, animals and feedingstuffs including information on foodborne outbreaks and antimicrobial resistance in zoonotic agents in 2012. 2012. Belgium.

EFSA (European Food Safety Authority). The Report referred to in Article 9 of Directive 2003/99/EC. Trends and sources of zoonoses and zoonotic agents in humans, foodstuffs, animals and feedingstuffs including information on foodborne outbreaks and antimicrobial resistance in zoonotic agents in 2013. 2013. Belgium.

EFSA (European Food Safety Authority). The European Union summary report on trends and sources of zoonoses, zoonotic agents and food-borne outbreaks in 2013. EFSA Journal. 2015a;13 1:3991, 165 pp.

EFSA (European Food Safety Authority). The European Union summary report on trends and sources of zoonoses, zoonotic agents and food-borne outbreaks in 2014. EFSA Journal. 2015b;13 12:4329, 191 pp.

Eichenberger RM, Stephan R, Deplazes P. Increased sensitivity for the diagnosis of *Taenia saginata* cysticercus infection by additional heart examination compared to the EU-approved routine meat inspection. Food Control. 2011;22(6):989-92.

Eichenberger RM, Lewis F, Gabriël S, Dorny P, Torgerson PR, Deplazes P. Multi-test analysis and model-based estimation of the prevalence of *Taenia saginata* cysticercus infection in naturally infected dairy cows in the absence of a 'gold standard' reference test. Int J Parasitol. 2013;43(10):853-9.

Embætti landlæknis (Directorate of Health). Farsóttaskýrslur 2013–2014. Tilkynningarskyldir sjúkdómar Farsóttagreining Sögulegar upplýsingar. Embætti landlæknis, sóttvarnalæknir. 2015. http://www.landlaeknir.is/servlet/file/store93/item28142/Fars%C3%B3ttask%C3%BDrslur_2013-2014_LOKA_24.11.2015.pdf. Accessed Mar 2017.

Esquivel A, Diaz-Otero F, Gimenez-Roldan S. Growing frequency of neurocysticercosis in Madrid (Spain). Neurología. 2005;20(3):116-20.

EVIRA (Finnish Food Safety Authority). Eräiden eläintautien esiintyminen Suomessa 2015. 2015. https://www.evira.fi/globalassets/elaimet/elainten-terveys-ja-elaintaudit/elaintaudit/tautitilasto-2015/liitea_taulukot-a1-a8-elaintautien-esiintymisesta-suomessa-2015.pdf. Accessed Feb 2017.

Faria F, Gama K, Correia MI, Faro Silva GF, Escórcia S, Vieira JJ, Câmara M, Duarte R, Alberto C, Nóbrega JJ. Um encontro inesperado… Medicina Interna. Portuguese Journal of Internal Medicine. Livro de resumos. XXI Congresso Nacional de Medicina Interna. 2015; 22 (Edição Especial).

Fernández-Aranda F, Solano R, Badía A, Jiménez-Murcia S. Binge eating disorder onset by unusual parasitic intestinal disease: a case-report. Int J Eat Disord. 2001;30(1):107-9.

Fernandez-Dominguez J, Gabaldon-Torres L, Salas-Felipe J, Aguilar-Amat-Prior MJ, Abenza-Abildua MJ, Arpa J. Ten years of neurocysticercosis at la paz unversity hospital in madrid (spain). European Journal of Neurology. 2007; 14(Suppl. 1);32–163.

Fernández-Rodríguez R, Ozaita G, Viso A, García-Mata R, Gómez A, Fernández O. Active and inactive forms of cerebral cysticercosis. Study of 10 cases. Rev Clin Esp. 1991;188(8):409-11.

Ferreira M, Brito MJ, Vieira JP, Salgueiro AB, Machado MC. Neurocisticercose em idade pediátrica. Acta Pediatr. Port. 2006;2(37):48-55.

Flores P, Serrão Neto A, João Xavier M, Durães F, Martins Palminha JM. Parasitoses Sistémicas. Acta Pediatr. Port. 2001;4(32):217-24.

Freire LMR. Acompanhamento da inspeção sanitária no matadouro Carne de Vinhais. Relatório Final de Estágio. Licenciatura em Medicina Veterinária. Universidade de Trás-os-Montes e Alto Douro. 2005;54pp.

FSA Scotland (Food Standards Agency Scotland). Verification of cold treatment of *C. bovis* in cold stores. Letter by Food Standards Agency Scotland to Lead Food Officers. 2008. https://www.food.gov.uk/sites/default/files/multimedia/pdfs/enforcement/enfs08019.pdf. Accessed Feb 2017.

FSAI (Food Safety Authority of Ireland). Food Safety Implications of Land-spreading Agricultural, Municipal and Industrial Organic Materials on Agricultural Land used for Food Production in Ireland. 2008. https://www.fsai.ie/WorkArea/DownloadAsset.aspx?id=8226. Acessed Mar 2017.

García Vallejo TB. Endoparasitosis del porcino ibérico en extremadura (España): Epidemiología y control. Tesis Doctoral en Medicina Veterinaria y Zootecnia. Universidad de Extremadura. 1999.

García-Castro (2003), cited in EFSA (European Food Safety Authority). Opinion of the Scientific Panel on Biological Hazards on “Risk assessment of a revised inspection of slaughter animals in areas with low prevalence of *Cysticercus*". EFSA Journal. 2004; 176:1-24.

Geerts S. *Taenia saginata*: an eternal problem?. Verh K Acad Geneeskd Belg. 1990;52(6):537-63.

Geerts S. *Taenia saginata* knaagt aan kwaliteit van rundvlees. Agricontact. 1992;236(8):1-6.

Gómez-Morales MA, Ludovisi A, Amati M, Pozio E. Serological diagnosis of human cysticercosis in Italy from 2001 to 2014. Proceedings of the 3rd CYSTINET Working Group Meeting, 12-13 May 2015, Rome, Italy.

Grande R, Ranzi ML, Restelli A, Maraschini A, Perego L, Torresani E. Intestinal parasitosis prevalence in outpatients and inpatients of Ca Granda IRCCS Foundation - Ospedale Maggiore Policlinico of Milan: data comparison between 1984-1985 and 2007-2009. Infez Med. 2011;19(1):28-38.

Gualdieri L, Rinaldi L, Petrullo L, Morgoglione ME, Maurelli MP, Musella V, et al. Intestinal parasites in immigrants in the city of Naples (southern Italy). Acta Trop. 2011;117(3):196-201.

Guidetti C, Ricci L, Vecchia L. Prevalence of intestinal parasitosis in Reggio Emilia (Italy) during 2009. Infez Med. 2010;18(3):154-61.

Halfon P, Penaranda G, Belgodere O. *Taenia* in the gastrointestinal tract after "figatellu" ingestion. Clin Res Hepatol Gastroenterol. 2014;38(3):243-4.

Hallanvuo S, Johansson T. Elintarvikkeiden mikrobiologiset vaarat. Eviran julkaisuja 1/2010. 2010, p.175.

Hernandez E, Cavallo JD, Debuysere H, Fiorina JC, Garrabe E. Prévalence des parasitoses digestives asymptomatiques: mise en évidence lors des examens d'aptitude aux emplois de l'alimentation. Bulletin epidemiologique hebdomadaire. 1997;14:61, 4 réf.

Hill AA, Horigan V, Clarke KA, Dewe TCM, Staerk KDC, O'Brien S, et al. A qualitative risk assessment for visual-only post-mortem meat inspection of cattle, sheep, goats and farmed/wild deer. Food Control. 2014;38:96-103.

Hoffman A, Jung M. Nudeln im Kolon. Endoskopie Heute. 2004;17 P9.

Ilsøe B, Kyvsgaard NC, Nansen P, Henriksen SA. Bovine cysticercosis in Denmark. A study of possible causes of infection in farms with heavily infected animals. Acta Vet Scand. 1990;31(2):159-68.

InVS (Institut de Veille Sanitaire). Morbidité et mortalité dues aux maladies infectieuses d'origine alimentaire en France. Rapport Institut de Veille Sanitaire. 2003. https://sites.anses.fr/fr/system/files/private/Morbidite_mortalite_INVS_2004.pdf. Accessed Feb 2017.

Jansen F. Evaluation and impact assessment of post mortem detection techniques for bovine cysticercosis in Belgium. Proceedings of the 3rd CYSTINET Working Group Meeting, 12-13 May 2015, Rome, Italy.

Januário G, Fonseca L, Novais G, Correia M, Irañeta A, Roque P, Monteiro J, Reis N. Neurocisticercose, uma série de 15 casos clínicos. 31º Congresso Nacional da Sociedad Portuguesa de Neurocirurgia, 28-30 Maio 2015, Porto, Portugal.

Jiménez Jiménez FJ, Molina Arjona JA, Zancada F, Santos J, Roldán Montaud A, Fernández Ballesteros A. Etiology of late-onset epilepsy. A prospective study in an area of rural health care. Medicina clinica. 1990;94(14):521-4.

Julini M. The slaughterhouse as an epidemiologic observatory. Industrie Alimentari. 1993;32:1075.

Kortbeek T. 2015. Personal communication.

Lobo J, González-Castillo J, Abad M, Nuevo JA, Cubo P, Muñoz S. Neurocysticercosis in Spain: an emerging problem. European Journal of Internal Medicine. 2003; 14:S1-S159.

Lopez-Caleya JF, Contreras SN, Martin-Rodrigo L. *Taenia saginata*: An imported case. Rev Esp Enferm Dig. 2015;107 7:440-1.

López-Vélez R, Huerga H, Turrientes MC. Infectious diseases in immigrants from the perspective of a tropical medicine referral unit. Am J Trop Med Hyg. 2003;69(1):115-21.

Lorenz J. The epidemiology of *Taenia saginata* taeniasis. Angew Parasitol. 1992;33(1):23-31.

Lortholary C, Lortholary O, Lapierre J. Cerebral cysticercosis epidemiology in France. Médecine et Maladies Infectieuses. 1990;20(2):115-116.

Martines H, Fanciulli E, Menardo G. Incidental video-capsule diagnosis of small-bowel *Taenia saginata* in a patient with recurrent hemorrhage due to angiodysplasias. Endoscopy. 2006;38(Suppl. 2):E35.

Más-Sesé G, Vives-Pinera I, Fernández-Barreiro A, Martínez-Lage JF, Martínez-Salcedo E, Alarcón-Martínez H, et al. A descriptive study of neurocysticercosis in a tertiary care hospital. Rev Neurol. 2008;46(4):194-6.

Masucci L, Graffeo R, Bani S, Bugli F, Boccia S, Nicolotti N, et al. Intestinal parasites isolated in a large teaching hospital, Italy, 1 May 2006 to 31 December 2008. Euro Surveill. 2011;16 24.

Mercky P, Gonzalez JM, Ah-Soune P. An unusual gastric encounter: a beef tapeworm diagnosed by gastroscopy. Endoscopy. 2014;46(Suppl. 1) UCTN:E36-7.

Minciullo PL, Spagnolo EV, Cascio A, Cardia G, Gangemi S. Fatal anaphylactic shock and *Taenia solium* infestation: a possible link? Ann Allergy Asthma Immunol. 2009;103(5):449-50.

Minguito Parra C, Duca A, Escamilla Fernandez N, Sollet Galean A, Pelaez Hidalgo A, Garcia Roch C, et al. Immigration and neurocysticercosis. A study from Spain. European Journal of Internal Medicine 20S. 2009;S1–S283.

Ministero della Salute, Dipartimento della Qualità Direzione Generale Programmazione Sanitaria (2011), cited in Zammarchi L, Strohmeyer M, Bartalesi F, Bruno E, Munoz J, Buonfrate D, et al. Epidemiology and management of cysticercosis and *Taenia solium* taeniasis in Europe, systematic review 1990–2011. PLoS One. 2013;8(7):e69537.

Möbius G. Epidemiologic studies of *C. bovis* and *T. saginata* infections in eastern and western Germany. Dtsch Tierarztl Wochenschr. 1993;100(3):110-4.

Monge-Maillo B, Jiménez BC, Pérez-Molina JA, Norman F, Navarro M, Pérez-Ayala A, et al. Imported infectious diseases in mobile populations, Spain. Emerg Infect Dis. 2009;15(11):1745-52.

Monteiro LASM. Neurocysticercosis in the North of Portugal. Arq Neuropsiquiatr. 1995;53 3-A.

Morgado C, Gomes LB, de Campos JG. Neurocysticercosis. An imaging analysis of 35 cases. [Acta Med Port.](https://www.ncbi.nlm.nih.gov/pubmed/8073900) 1994;7(5):269-75.

MSSSI (Ministerio de Sanidad, Servicios Sociales e Igualdad). Instituto de Información Sanitaria. 2016. Registro de altas - CMBD estatal Altas hospitalarias CMBD-H. Spain.

Nagel-Kohl. Results of Meat Inspection Concerning the Prevalence of Cysticercosis in Germany. Proceedings of the 1st CYSTINET Working Group Meeting, 6-7 Jul 2014, Evora, Portugal.

National inpatient diagnosis register. Denmark. 2015. Personal communication.

NIJZ (Nacionalni inštitut za javno zdravje). Epidemiological surveillance of infectious diseases - annual report 1997. 1998. http://www.nijz.si/sl/epidemiolosko-spremljanje-nalezljivih-bolezni-letna-in-cetrtletna-porocila. Accessed Feb 2017.

NIJZ (Nacionalni inštitut za javno zdravje). Epidemiological surveillance of infectious diseases - annual report 1998. 1999. http://www.nijz.si/sl/epidemiolosko-spremljanje-nalezljivih-bolezni-letna-in-cetrtletna-porocila. Accessed Feb 2017.

NIJZ (Nacionalni inštitut za javno zdravje). Epidemiological surveillance of infectious diseases - annual report 1999. 2000. http://www.nijz.si/sl/epidemiolosko-spremljanje-nalezljivih-bolezni-letna-in-cetrtletna-porocila. Accessed Feb 2017.

NIJZ (Nacionalni inštitut za javno zdravje). Epidemiological surveillance of infectious diseases - annual report 2000. 2001. http://www.nijz.si/sl/epidemiolosko-spremljanje-nalezljivih-bolezni-letna-in-cetrtletna-porocila. Accessed Feb 2017.

NIJZ (Nacionalni inštitut za javno zdravje). Epidemiological surveillance of infectious diseases - annual report 2001. 2002. http://www.nijz.si/sl/epidemiolosko-spremljanje-nalezljivih-bolezni-letna-in-cetrtletna-porocila. Accessed Feb 2017.

NIJZ (Nacionalni inštitut za javno zdravje). Epidemiological surveillance of infectious diseases - annual report 2002. 2003. http://www.nijz.si/sl/epidemiolosko-spremljanje-nalezljivih-bolezni-letna-in-cetrtletna-porocila. Accessed Feb 2017.

NIJZ (Nacionalni inštitut za javno zdravje). Epidemiological surveillance of infectious diseases - annual report 2003. 2005a. http://www.nijz.si/sl/epidemiolosko-spremljanje-nalezljivih-bolezni-letna-in-cetrtletna-porocila. Accessed Feb 2017.

NIJZ (Nacionalni inštitut za javno zdravje). Epidemiological surveillance of infectious diseases - annual report 2004. 2005b. http://www.nijz.si/sl/epidemiolosko-spremljanje-nalezljivih-bolezni-letna-in-cetrtletna-porocila. Accessed Feb 2017.

NIJZ (Nacionalni inštitut za javno zdravje). Epidemiological surveillance of infectious diseases - annual report 2005. 2006. http://www.nijz.si/sl/epidemiolosko-spremljanje-nalezljivih-bolezni-letna-in-cetrtletna-porocila. Accessed Feb 2017.

NIJZ (Nacionalni inštitut za javno zdravje). Epidemiological surveillance of infectious diseases - annual report 2006. 2007. http://www.nijz.si/sl/epidemiolosko-spremljanje-nalezljivih-bolezni-letna-in-cetrtletna-porocila. Accessed Feb 2017.

NIJZ (Nacionalni inštitut za javno zdravje). Epidemiological surveillance of infectious diseases - annual report 2007. 2008. http://www.nijz.si/sl/epidemiolosko-spremljanje-nalezljivih-bolezni-letna-in-cetrtletna-porocila. Accessed Feb 2017.

NIJZ (Nacionalni inštitut za javno zdravje). Epidemiological surveillance of infectious diseases - annual report 2008. 2009. http://www.nijz.si/sl/epidemiolosko-spremljanje-nalezljivih-bolezni-letna-in-cetrtletna-porocila. Accessed Feb 2017.

NIJZ (Nacionalni inštitut za javno zdravje). Epidemiological surveillance of infectious diseases - annual report 2009. 2010. http://www.nijz.si/sl/epidemiolosko-spremljanje-nalezljivih-bolezni-letna-in-cetrtletna-porocila. Accessed Feb 2017.

NIJZ (Nacionalni inštitut za javno zdravje). Epidemiological surveillance of infectious diseases - annual report 2010. 2011. http://www.nijz.si/sl/epidemiolosko-spremljanje-nalezljivih-bolezni-letna-in-cetrtletna-porocila. Accessed Feb 2017.

NIJZ (Nacionalni inštitut za javno zdravje). Epidemiological surveillance of infectious diseases - annual report 2011. 2012. http://www.nijz.si/sl/epidemiolosko-spremljanje-nalezljivih-bolezni-letna-in-cetrtletna-porocila. Accessed Feb 2017.

NIJZ (Nacionalni inštitut za javno zdravje). Epidemiological surveillance of infectious diseases - annual report 2012. 2013. http://www.nijz.si/sl/epidemiolosko-spremljanje-nalezljivih-bolezni-letna-in-cetrtletna-porocila. Accessed Feb 2017.

NIJZ (Nacionalni inštitut za javno zdravje). Epidemiological surveillance of infectious diseases - annual report 2013. 2014. http://www.nijz.si/sl/epidemiolosko-spremljanje-nalezljivih-bolezni-letna-in-cetrtletna-porocila. Accessed Feb 2017.

NIJZ (Nacionalni inštitut za javno zdravje). Epidemiological surveillance of infectious diseases - annual report 2014. 2015. http://www.nijz.si/sl/epidemiolosko-spremljanje-nalezljivih-bolezni-letna-in-cetrtletna-porocila. Accessed Feb 2017.

NVI (Norwegian Veterinary Institute). Årsrapport 2011 for Veterinærinstituttet. 2012. http://www.nsd.uib.no/polsys/data/filer/aarsmeldinger/AN_2011_55790.pdf. Accessed Jan 2017.

NVWA (Nederlandse Voedsel-en Warenautoriteit). Advies over de risico's van tuberculose en cysticercose in vleeskalveren bij aangepast keuringsbeleid. Advies van de directeur bureau Risicobeoordeling & onderzoeksprogrammering aan de minister van VWS en de staatssecretaris van EZ. 2013.

Overbosch D, Oosterhuis JW, Kortbeek LM, Garcia-Albea E. Neurocysticercosis in Europe. In: Craig P, Pawlowski Z, editors. Cestode Zoonoses: Echinococcosis and Cysticercosis An emergent and global problem. Amsterdam: IOS press NATO Science Series; 2002. p. 33–40.

Paolantonio P, Rengo M, Iafrate F, Martino G, Laghi A. Diagnosis of *Taenia saginata* by MR enterography. AJR Am J Roentgenol. 2006;187(2):W238.

Pezzoli A, Fusetti N, Pizzo E. Capsule endoscopy diagnosis of intestinal *Taenia*. Gastrointest Endosc. 2015 (Epub 2015 Aug).

Poirè G, Sassetti M, Magi M. Cisticercosi bovina. I riscontri della parassitosi negli impianti di macellazione del Chiavarese. Documenti Veterinari. 1994;5:55-57.

Puschmann A, Cronqvist J, Maly P, Englund E, Pessah-Rasmussen H. Neurocysticerkos som orsak till epileptiskt anfall. Infektion med svinbandmask finns sporadiskt i Skandinavien. Läkartidningen. 2006;12(103):938-942.

Rakuša. Human cysticercosis in Slovenia. Laboratory for Parasitology, Institute of Microbiology and Immunology, Faculty of Medicine. http://www.arhimed.co.uk/arhimed/poglej.asp?id=80. Accessed Feb 2017.

Ramos JM, Masia M, Padilla S, Escolano C, Bernal E, Gutierrez F. Imported and non-imported diseases in the immigrant population. A decade of experience from an infectious diseases unit. Enferm Infecc Microbiol Clin. 2011;29(3):185-92.

RIVM (Rijksinstituut voor Volksgezondheid en Milieu). Staat van zoönosen 2012. RIVM Rapport. 2013. http://www.rivm.nl/dsresource?objectid=936c4d80-27bb-47d5-a27a-483680804bd7&type=org&disposition=inline. Accessed Feb 2017.

Roberts MT, Lever AM. An analysis of imported infections over a 5-year period at a teaching hospital in the United Kingdom. Travel Med Infect Dis. 2003;1(4):227-30.

Roca C, Balanzó X, Fernández-Roure JL, Sauca G, Savall R, Gascón J, et al. Imported diseases in African immigrants in Spain: study of 1,321 patients. [Med Clin (Barc).](https://www.ncbi.nlm.nih.gov/pubmed/12433338) 2002;119(16):616-9.

Roca C, Gascón J, Font B, Pujol T, Valls ME, Corachán M. Neurocysticercosis and population movements: analysis of 23 imported cases in Spain. Eur J Clin Microbiol Infect Dis. 2003;22(6):382-4.

Rodríguez Guardado A, Gómez E, Rodríguez Pérez M, Sempere A, López-Roger Roger R, Suárez Leiva P, et al. P540 Cysticercosis: correlation between serological and radiological diagnosis. International Journal of Antimicrobial Agents. 2007;29:S120-S1.

Rousseau MC, Guillotel B, Delmont J. Neurocysticercosis in the South-East of France 1988-1998. Presse Med. 1999;28(39):2141-4.

Ruiz S, García-Vázquez E, Picazo R, Hernández A, Herrero JA, Gómez J. Neurocysticercosis in Murcia (Spain). Rev Clin Esp. 2011;211(3):133-8.

Sánchez Martínez P. Diagnóstico de procesos patológicos en ungulados domésticos sacrificados en matadero en el sureste de España. Tesis doctoral. Universidad de Murcia. Departamento de Anatomía y anatomía patológica comparadas. 2013.

Schmutzhard E, Auer H. 2017. Personal communication.

SIM (Sistema de Información Microbiológica). Instituto de Salud Carlos III, Spain. http://www.isciii.es/ISCIII/es/contenidos/fd-servicios-cientifico-tecnicos/fd-vigilancias-alertas/fd-sistema-informacion-microbiologica/informes-generales.shtml. Accessed Feb 2017.

SIM (Sistema de Información Microbiológica), cited in Fos Claver S, Vendrell Blay E, Minardi Mitre R, Morales Suárez-Varela MM, Llopis González A. Enfermedades parasitarias de origen alimentario más frecuentes en España: incidencia y comparación con las de origen vírico y bacteriano. Ars Pharmaceutica. 2000;41(3):293-305.

SIM (Sistema de Información Microbiológica), cited in ISP (Instituto de Salud Pública de Navarra). Boletín Informativo. 2002; 22.

Šoba B. 2015. Personal communication.

Stensvold CR. 2017. Personal communication.

Tamburrini A, Gomez Morales MA, Pozio E. Development of an immunoenzyme test for the diagnosis of human cysticercosis using a heterologous antigen. Parassitologia. 1995;37(2-3):195-8.

Tomaso H, Dierich MP, Allerberger F. Helminthic infestations in the Tyrol, Austria. Clin Microbiol Infect. 2001;7(11):639-41.

Treurniet, HF. Zoönose in Nederland: een inventarisatie. Infectieziekten Bulletin. 1993;4(5):92-97.

UVHVVR (Administration of the Republic of Slovenia for Food Safety, Veterinary Sector and Plant Protection). Letno poročilo o zoonozah in povzročiteljih zoonoz, 2014. 2015. http://www.uvhvvr.gov.si/fileadmin/uvhvvr.gov.si/pageuploads/DELOVNA_PODROCJA/Zivila/zoonoze/POROCILO_ZOONOZE_2014.pdf. Accessed Feb 2017.

Valadas E, Badura R, Marques T, Neno M, Boura M, Sutre AF, et al. A case of imported neurocysticercosis in Portugal. J Infect Dev Ctries. 2015;9(1):114-7.

Van Beurden A, Ottow RT, Van Oerle MC. An unusual ultrasound diagnosis of right lower quadrant abdominal pain. Gut. 2008;57(4):515.

Van der Logt PB, Gottstein B. Unidentified parasitic cysts in cattle. Vet Rec. 2000;146(21):610-2.

Van Roermund JG, Klaase JM. Diagnostic image (160). A woman with a tapeworm in a stoma. *Taenia saginata*. Ned Tijdschr Geneeskd. 2003;147(41):2020.

Van Tiggelen P, Danse E. Images in clinical radiology. Intestinal taeniasis. Journal belge de radiologie. 1998;81(1):22.

Vergles-Rataj A, Krt B, Starič J, Ježek J, Dorny P, Gabriël S, Šoba B. Outbreak of bovine cysticercosis on a cattle farm in Slovenia in 2015 – a case report. Proceedings of the 1st CYSTINET International conference. Taeniosis and cysticercosis: a one health challenge. 3-4 November 2015, Belgrade, Serbia.

Vieira-Pinto M, Pires I, Freire L, Dorny P. Muscular calcification in dry-meat ham from a pig raised in an outdoor farm - A challenge poster: Try to find the possible diagnosis. Proceedings of the 3rd CYSTINET Working Group Meeting, 12-13 May 2015, Rome, Italy.

Vilhena M, Torgal J, Dias S. Cysticercosis in Portugal: is there a probability for reemergence? Trop Med Int Health. 2007;12(Suppl. 1):147–255.

Vilhena M, Fonseca AG, Marques da Silva JR, Dias SS, Torgal J. Characterizing human cysticercosis in Portugal 2006-2013. Proceedings of the 1st CYSTINET International conference. Taeniosis and cysticercosis: a one health challenge. 3-4 November 2015, Belgrade, Serbia.

Vilhena M. 2017. Personal communication.

Villafruela M, de Manuel J, Sandoval A. Taeniasis in the 21st century. Rev Esp Enferm Dig. 2009;101(2):149.

Vuylsteke P, Bertrand C, Verhoef GE, Vandenberghe P. Case of megaloblastic anemia caused by intestinal taeniasis. Ann Hematol. 2004;83(7):487-8.

Zammarchi L, Veneruso G, Strohmeyer M, Mantella A, Bartalesi F, Bianchi L et al. *Taenia solium* seroprevalence in immigrant and foreign adopted children in florence, Italy. Trop Med Int Health. 2011;16(Suppl. 1):97-384.

1. Country not specified. [↑](#footnote-ref-1)
2. Most likely natives of the country where diagnosis was made. [↑](#footnote-ref-2)
3. Up to 14 levels of diagnosis included [↑](#footnote-ref-3)
4. Year of publication [↑](#footnote-ref-4)
5. Year of publication [↑](#footnote-ref-5)
6. The 53 diagnosed cases were mostly from Portuguese-speaking African countries. As no further details were available the 53 cases were classified here as imported cases. [↑](#footnote-ref-6)
7. Most of them were from Cape-Verde. As no further details were available, the 15 cases were classified as imported. [↑](#footnote-ref-7)
8. It is not known if some cases published by Más-Sesé, et al. (2008) and Ruiz et al. (2011) could be duplicates as were diagnosed in the same hospital and some years overlap. [↑](#footnote-ref-8)
9. Information based on total condemnations only. [↑](#footnote-ref-9)
10. Large variation in prevalence among districts (1.96-33.33%) [↑](#footnote-ref-10)
11. Based on Bayesian approach estimated prevalence of 16.5% [↑](#footnote-ref-11)
